# Hepatitis E Virus in Water Environments: A Systematic Review and Meta-analysis

**DOI:** 10.1007/s12560-022-09530-3

**Published:** 2022-08-29

**Authors:** G. R. Takuissu, S. Kenmoe, L. Ndip, J. T. Ebogo-Belobo, C. Kengne-Ndé, D. S. Mbaga, A. Bowo-Ngandji, M. G. Oyono, R. Kenfack-Momo, S. Tchatchouang, J. Kenfack-Zanguim, R. Lontuo Fogang, E. Zeuko’o Menkem, G. I. Kame-Ngasse, J. N. Magoudjou-Pekam, S. Nkie Esemu, C. Veneri, P. Mancini, G. Bonanno Ferraro, M. Iaconelli, E. Suffredini, G. La Rosa

**Affiliations:** 1 Centre for Food, Food Security and Nutrition Research, Institute of Medical Research and Medicinal Plants Studies, Yaoundé, Cameroon; 2 Department of Microbiology and Parasitology, University of Buea, Buea, Cameroon; 3 Medical Research Centre, Institute of Medical Research and Medicinal Plants Studies, Yaoundé, Cameroon; 4 Epidemiological Surveillance, Evaluation and Research Unit, National AIDS Control Committee, Douala, Cameroon; 5 Department of Microbiology, The University of Yaounde I, Yaoundé, Cameroon; 6 Centre for Research on Health and Priority Pathologies, Institute of Medical Research and Medicinal Plants Studies, Yaoundé, Cameroon; 7 Department of Biochemistry, The University of Yaounde I, Yaoundé, Cameroon; 8 Scientific Direction, Centre Pasteur du Cameroun, Yaoundé, Cameroon; 9 Department of Animal Biology, University of Dschang, Dschang, Cameroon; 10 Department of Biomedical Sciences, University of Buea, Buea, Cameroon; 11 Department of Environment and Health, Istituto Superiore di Sanità, Rome, Italy; 12 Department of Food Safety, Nutrition and Veterinary Public Health, Istituto Superiore di Sanità, Rome, Italy

**Keywords:** Hepatitis E, HEV, Water matrices, Prevalence

## Abstract

Hepatitis E virus (HEV) is responsible for acute hepatitis in humans, through foodborne, zoonotic, and waterborne transmission routes. This study aimed to assess the prevalence of HEV in water matrices. Six categories were defined: untreated and treated wastewater, surface water (river, lake, and seawater), drinking water, groundwater, and other water environments (irrigation water, grey water, reservoir water, flood water, and effluent of pig slaughterhouse). We searched PubMed, Web of Science, Global Index Medicus, and Excerpta Medica Database. Study selection and data extraction were performed by at least two independent investigators. Heterogeneity (*I*^2^) was assessed using the *χ*^2^ test on the Cochran *Q* statistic and *H* parameter. Sources of heterogeneity were explored by subgroup analysis. This study is registered with PROSPERO, number CRD42021289116. We included 87 prevalence studies from 58 papers, 66.4% of which performed in Europe. The overall prevalence of HEV in water was 9.8% (95% CI 6.4–13.7). The prevalence was higher in untreated wastewater (15.1%) and lower in treated wastewater (3.8%) and in drinking water (4.7%). In surface water, prevalence was 7.4%, and in groundwater, the percentage of positive samples, from only one study available, was 8.3%. Overall, only 36.8% of the studies reported the genotype of HEV, with genotype 3 (HEV-3) prevalent (168 samples), followed by HEV-1 (148 sample), and HEV-4 (2 samples). High-income countries were the most represented with 59/87 studies (67.8%), while only 3/87 (3.5%) of the studies were performed in low-income countries. The overall prevalence obtained of this study was generally higher in industrialized countries. Risk of bias was low in 14.9% of the studies and moderate in 85.1%. The results of this review showed the occurrence of HEV in different waters environments also in industrialized countries with sanitation and safe water supplies. While HEV transmission to humans through water has been widely demonstrated in developing countries, it is an issue still pending in industrialized countries. Better knowledge on the source of pollution, occurrence, survival in water, and removal by water treatment is needed to unravel this transmission path.

**Graphical Abstract:**

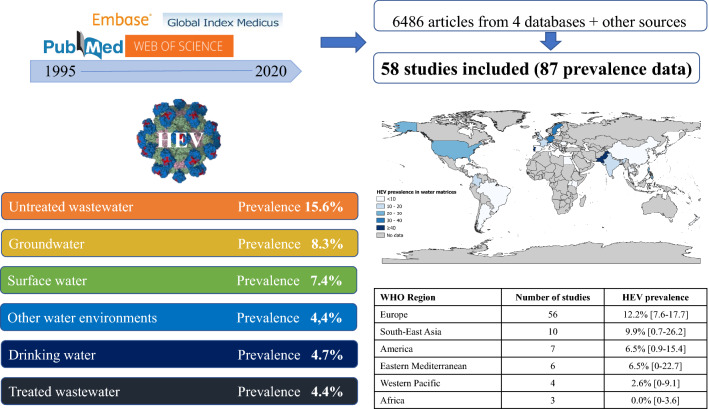

**Supplementary Information:**

The online version contains supplementary material available at 10.1007/s12560-022-09530-3.

## Introduction

Hepatitis E is a liver infection caused by the hepatitis E virus (HEV), a single-stranded RNA virus belonging to the *Hepeviridae* family; species *Orthohepevirus* A of the genus *Orthohepevirus* (Capai et al., 2018). HEV is an enteric virus responsible for both acute and chronic disorders mainly in the liver (Pischke et al., 2017). It can also cause neurological manifestations in both acutely and chronically HEV-infected patients (Cheung et al., 2012; Lhomme et al., 2021), kidneys (Kamar et al., 2012), pancreas, and thrombocytes (Aggarwal, 2011). The WHO (2021) noted about 20 million new cases of HEV infections each year, with over 3 million symptomatic cases. This poses the problem of prevention by acting primarily at the transmission level (World Health Organization, 2021). In the vast majority of infected individuals, HEV infection results in a self-limited, acute disease; however, acute infection can become chronic in rare cases, almost exclusively associated to genotype 3 in immunocompromised patients, including those that have undergone solid organ or stem cell transplantation (Damiris et al., 2022; Marion et al., 2016). Members of *Orthohepevirus A* have been assigned to eight different genotypes, G1–G8 (Purdy et al., 2017). Of these, genotypes G1–G4 are most commonly associated with HEV infection in humans, and are responsible for different epidemiologic and clinical characteristics in industrialized and non-industrialized countries. Hepatitis E usually occurs as large outbreaks in areas where the virus is endemic (genotype 1 in Asia and Africa, genotype 2 in Mexico, and genotype 4 in Taiwan and China) or as sporadic autochthonous cases in industrialized countries (genotype 3) (CDC, 2020). While genotypes 1 and 2 have been found only in humans, genotypes 3 and 4 circulate in several animals including pigs, wild boars, and deer without causing any disease, and occasionally infect humans (World Health Organization, 2021).

The main route of transmission of HEV is the faecal–oral route, which can be via animals, food, and water. In countries with limited access to essential water, sanitation, hygiene and health services, inadequate disposal and treatment of sewage, and contamination of drinking and irrigation water lead to epidemics. Indeed, there is considerable epidemiological evidence of waterborne HEV transmission, especially in Southern/South-East/Central Asia and North-West Africa (Hakim et al., 2017; Khuroo et al., 2016; Singh et al., 2016). Methods for investigating waterborne outbreaks of hepatitis E, and measures for their prevention and control were published by the World Health Organization (WHO) in a technical report in 2014 (World Health Organization, 2014). In areas with better water supply and sanitation, only sporadic cases occur, caused mainly by genotype 3, acquired through zoonotic HEV infections by eating undercooked pig flesh, raw liver, and sausages. Indeed, a systematic review on transmission routes and risk factors for autochthonous hepatitis E virus infection in Europe showed that although evidence points towards zoonotic transmission, multiple routes of transmission are likely to exist, which need to be further defined in order to implement appropriate preventive measures (Lewis et al., 2010). Similarly, Mrzljak et al. emphasized the need of a «One-Health» multidisciplinary collaboration, in the surveillance and control of this infection (Mrzljak et al., 2019), looking at the human, animal, and environmental interface.

Waterborne transmission, the most important mode of transmission in non-industrialized countries, has not been investigated in developed countries despite different studies demonstrated the occurrence of HEV in water environments. Waterborne transmission can be direct, by contact with contaminated drinking or recreational waters, or indirect, e.g. through contamination of shellfish production areas, or by irrigation of crops using contaminated waters. Moreover, a few studies pointed out that surface waters can be contaminated in the vicinity of pig production facilities in particular as a consequence of run-off waters and percolation events or following the agronomic use of pig slurry (Gentry-Shields et al., 2015; Steyer et al., 2011). Some authors have pointed out the current lack of knowledge on possible waterborne transmissions in developed countries (King et al., 2018; Van der Poel et al., 2018). Studies performed in France suggested that contaminated water could contribute to the epidemiology of HEV infection in this country (Mansuy et al., 2015, 2016).

A few systematic reviews on HEV have been published to date. Li et al. published a systematic review on the global epidemiology of hepatitis E virus infection and related risk factors (Li et al., 2020). A systematic review conducted only in Brazil collected and analysed data on HEV in humans, animals, and the environment from a One Health perspective indicating that HEV-3 is widespread in the country, and sanitary surveillance is essential in the national production of pigs (Moraes et al., 2021). Modiyinji et al. conducted a systematic review on the prevalence of HEV in animals in Africa, highlighting that some animals could be the reservoir of HEV, and suggesting the need of molecular epidemiological studies for investigating zoonotic transmission (Modiyinji et al., 2021). Narrative reviews have addressed the occurrence of HEV in food and aquatic systems (Treagus et al., 2021) but no systematic review has yet addressed the prevalence of HEV in water environments, despite several studies on different water matrices have been carried out in different regions of the world, in both developed and developing countries. The objective of this systematic review and meta-analysis was to assess the overall prevalence of HEV in water matrices worldwide.

## Materials and Methods

### Protocol and Registration

We performed this study in accordance with the PRISMA guidelines (Moher et al., 2009). The registration of the protocol was done in the International Prospective Register of Systematic Reviews (PROSPERO, no. CRD42021289116).

### Data Sources and Search Strategy

The searches were performed in four databases: PubMed, Excerpta Medica Database (Embase), Web of Science, and Global Index Medicus. Databases were searched for articles related to HEV and water matrices, published worldwide from inception until February 2022 (Table S1). A manual search was also conducted to browse reference lists of eligible articles.

### Study Selection

Studies were included if they met the following criteria: (a) written in English or French and (b) which contained data about the prevalence of HEV RNA in water matrices. The following studies were excluded: (a) systematic reviews, meta-analysis, comments, case reports, and case series, (b) no water matrix, (c) no primary data, (d) duplicate data, and (e) studies with < 10 samples.

### Data Extraction and Management

Data of included studies were extracted by investigators using a pre-designed Google data abstraction form. Two reviewers resolved discrepancies by jointly reviewing the study in question. If no consensus was reached, a third reviewer, unaware of prior determinations, functioned as an arbiter. The following data were extracted: name of the first author, year of publication, sampling period, sampling approach (probabilistic/non-probabilistic), number of sites (multicenter, monocenter or nationally representative), setting (urban/rural), timing of sample collection (prospectively/retrospectively), country, type of water matrices, methods of detecting HEV (conventional/real-time PCR), total number of samples, and number of sample positive for HEV. WHO region, United Nations Statistics Division (UNSD) region (Jian et al., 2022), and country income level (World Bank, 2022) were assigned to each included study according to the reported country.

### Quality Assessment

The tool developed by Hoy et al. for prevalence studies was adapted and used to assess the quality of the studies (Hoy et al., 2012). It included nine items: study’s target population representation, sampling representation, form of random selection, acceptable water matrix definition, validity and reliability of detection assay, mode of data collection, length of the study period, and reporting numerator(s) and denominator(s) for the prevalence of HEV. This allowed the included studies to be assessed for risk of bias (7–9 = low risk; 4–6 = moderate risk; 0–3 = high risk), rigour, and transparency (Table S2).

### Statistical Analysis

Pooled prevalences were calculated using a DerSimonian and Laird random-effects model meta-analysis (DerSimonian & Laird, 1986). For the calculation of pooled prevalence, the Freeman–Tukey Double arcsine transformation was implemented (Barendregt et al., 2013). Weighting according to the sample size was used to determine the size of the diamonds for plotting individual studies and pooled prevalence (Higgins et al., 2009). The 95% confidence interval (95% CI) for the individual studies and overall prevalence were calculated using the Clopper–Pearson method (Agresti & Coull, 1998; Newcombe, 1998). A prediction interval of values for future studies was estimated (Guddat et al., 2012; Higgins et al., 2009). Heterogeneity was assessed by the Cochrane *Q* statistical test and quantified by *I*^2^ values, assuming that the *I*^2^ values of 25%, 50%, and 75% represent low, moderate, and high heterogeneity, respectively (Higgins et al., 2003). Publication bias was assessed by Egger’s test and the funnel plot (Egger et al., 1997). Subgroup analyses were conducted according to sampling approach, setting, country, WHO and UNSD regions, country income level, water matrix, and HEV detection assay. To test the robustness of the overall results, studies with low risk of bias and those with process control (RNA concentration, extraction and/or amplification) were used for sensitivity analysis. A *p* value < 0.05 indicated a significant difference. R software version 4.1.0 was used to perform analyses (Borenstein et al., 2010; Schwarzer, 2007).

## Results

### Study Selection

**Fig. 1:**
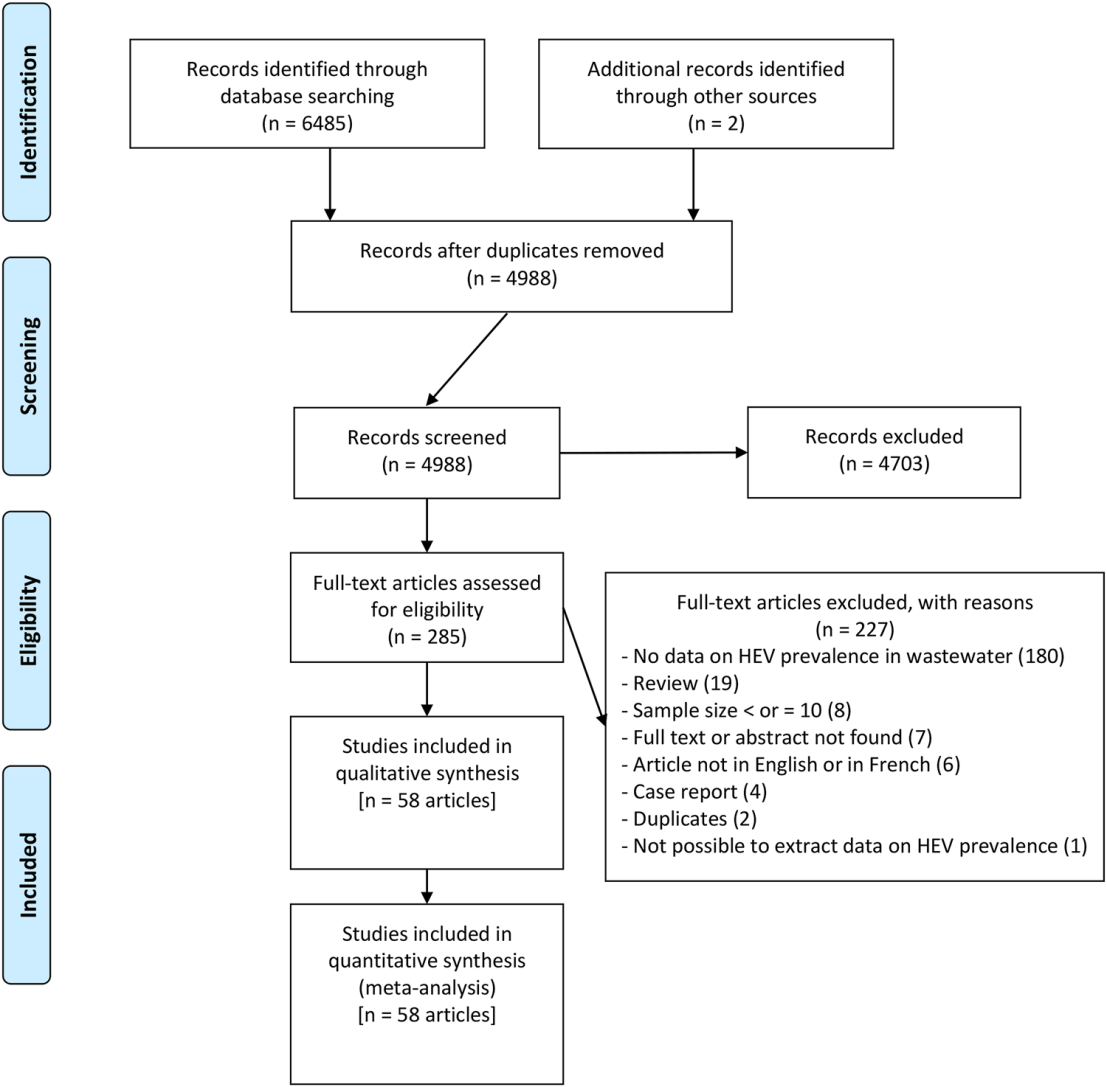
Screening diagram of prevalence of HEV in various water sources

After searching the databases, we obtained 6485 articles (Fig. 1). After eliminating 1499 duplicates, 4703 articles were further excluded for inappropriate titles or abstracts. A total of 285 articles were therefore assessed as eligible, from which 227 were excluded for reasons given in Table S3. Finally, a total of 58 articles were included in the qualitative and quantitative synthesis of this review after the application of the eligibility criteria defined (Ahmad et al., 2010; Alfonsi et al., 2018; Baez et al., 2017; Béji-Hamza et al., 2015; Beyer et al., 2020; Bisseux et al., 2018; Clemente-Casares et al., 2003; Cuevas-Ferrando et al., 2020; Di Profio et al., 2019; El-Esnawy, 2000; El-Esnawy et al., 1998; El-Senousy & Abou-Elela, 2017; Farkas et al., 2018; Fenaux et al., 2018; Hazam et al., 2010; Heldt et al., 2016; Howard et al., 2010; Iaconelli et al., 2015, 2017, 2020; Idolo et al., 2013; Ippagunta et al., 2007; Jothikumar et al., 1995, 2000; Kamel et al., 2011; Katukiza et al., 2013; Kokkinos et al., 2011; La Fauci et al., 2010; La Rosa et al., 2010, 2017, 2018; Li et al., 2014, 2017; Martínez Wassaf et al., 2014; Masclaux et al., 2013; Matos et al., 2018; Miura et al., 2016; Ngaosuwankul et al., 2013; Pina et al., 1998, 2000; Pisano et al., 2018; Prevost et al., 2015; Purpari et al., 2019; Rahmani et al., 2020; Ram et al., 2016; Randazzo et al., 2018; Rusiñol et al., 2020; Rutjes et al., 2009; Salvador et al., 2020; Smith et al., 2016; Steyer et al., 2011, 2015; Thongprachum et al., 2018; Vaidya et al., 2002; Verma & Arankalle, 2010; Vivek et al., 2013; Wang et al., 2020b; Williamson et al., 2011). Among these 58 articles, some were dealing with more than one water matrix, corresponding to 87 prevalence data.

### Study Characteristics

The characteristics of the included studies are listed in Tables S4 and S5. The studies were published between 1995 and 2020, and the water samples were collected between 1989 and 2019. The majority of studies performed non-probabilistic sampling approach (66/87, 75.9%) were prospective (86/87, 98.9%), monocenter (46/87, 52.9%), or multicenter (40/87, 46.0%). The most represented UNSD and WHO Regions were respectively Southern Europe 34/87 (39.1%) and Europe 56/87 (64.4%). The most represented country was Italy 18/87 (20.7%). High-income countries were the most represented 59/87 (67.8%). The water matrices were categorized into six groups, of which the most represented was untreated wastewater (40/87, 46.0%), followed by surface water (20/87, 23.0%), treated wastewater (11/87, 12.6%), drinking water (10/87, 11.5%), other water matrices (including grey water, flood water, seawater, pipe water, irrigation water, reservoir water, and effluents of pig slaughterhouse) (5/87, 5.8%), and groundwater (1/87, 1.2%). The majority of studies was at a moderate risk of bias (74/87, 85.1%), while 13/87 (14.9%) were at low risk of bias.

A considerable variability in the methods used to detect HEV was documented (Table S4) in all analytical phases:

i. Volume of water used, ranging from 0.01 to 100 L, depending on the water matrix;
ii. Method of viral concentration (e.g. polyethylene glycol precipitation, ultracentrifugation, filtration, organic flocculation, absorption-elution method, others);
iii. Method for RNA extraction (e.g. silica capture protocol with magnet beads, TRIzol reagent, column-based nucleic acid extraction kits, others);
iv. Genomic region used for detecting and/or typing HEV (ORF1, ORF2, ORF3, different combinations of the three ORF regions).

Moreover, only 47.1% of the prevalence studies reported the use of a process control to monitor the efficiency of the virus concentration/extraction steps.

The most commonly used diagnostic method was real-time PCR (60/87, 69.0%). Overall, only 36.8% of the studies reported the genotype of HEV, the most frequent was genotype 3 (HEV-3) (168 samples), followed by HEV-1 (148 samples), and HEV-4 (2 samples).

### HEV Prevalence in Water Matrices

**Fig. 2:**
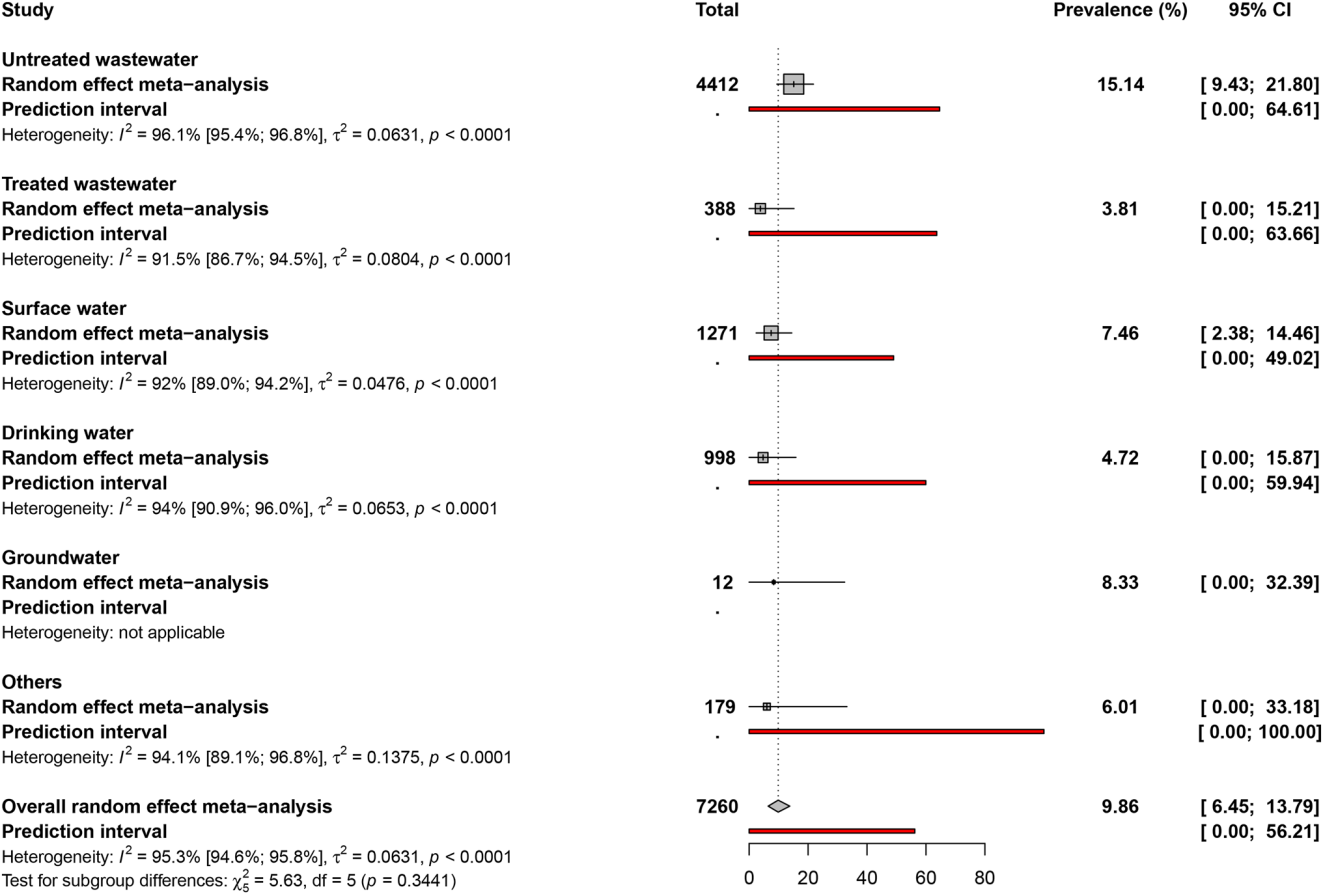
Global prevalence estimate of HEV in various water matrices

**Fig. 3:**
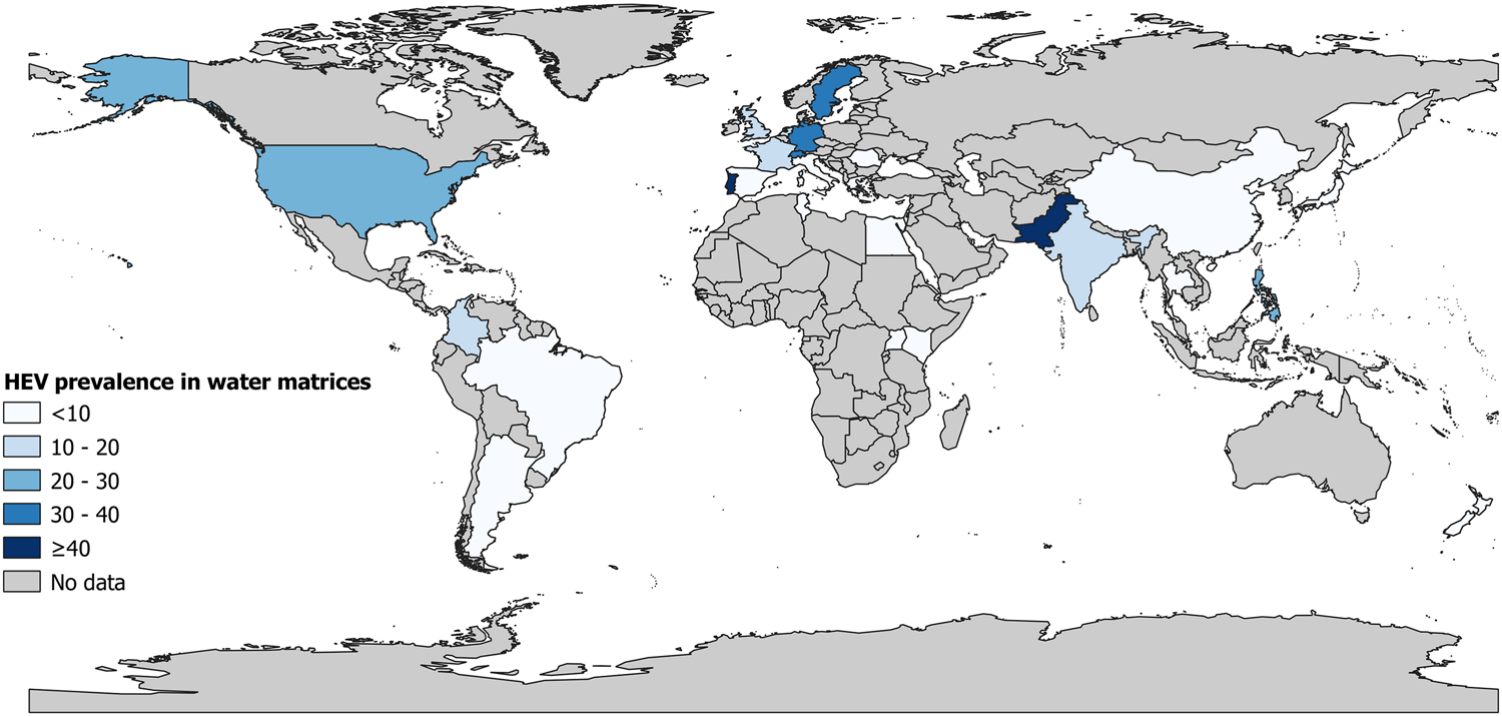
Global repartition of prevalence estimate of HEV in various water matrices

The HEV prevalence in water matrix, represented in Figs. 2, 3, and S1, was 9.8% (95% CI 6.4–13.7). According to the different water matrix groups, the prevalence varied from 3.8 to 15.1%. In order, the prevalence was 15.1% (95% CI 9.4–21.8) in untreated wastewater, 8.3% (95% CI 0.0–32.3) in groundwater, 7.4% (95% CI 2.3–14.4) in surface water, 6.0% (95% CI 0.0–33.1) in other water matrices, 4.7% (95% CI 0.0–15.8) in drinking water, and 3.8 (95% CI 0.0–15.2) in treated wastewater. Sensitivity analyses including only studies with a low risk of bias and studies that applied a control of the analytical process (concentration, extraction and/or amplification of RNA) did not significantly influence the overall results (Table 1).

**Table 1:** Summary of global meta-analysis results for prevalence of hepatitis E virus in different water matrices divided by risk of bias

Water matrix	Prevalence (%) [95%CI]	95% Prediction interval	No of studies	No of samples	^¶^H [95%CI]	^§^I^2^ [95%CI]	*P* heterogeneity
Untreated wastewater							
Overall	15.1 [9.4–21.8]	[0–64.6]	40	4412	5.1 [4.7–5.6]	96.1 [95.4–96.8]	< 0.001
Low risk of bias	9.7 [0.9–24.7]	[0–75.1]	8	1886	6.1 [5.1–7.4]	97.3 [96.2–98.2]	< 0.001
Process controlled	17.4 [7.8–29.6]	[0–75.8]	16	2414	5.8 [5.1–6.6]	97 [96.1–97.7]	< 0.001
Treated wastewater							
Overall	3.8 [0–15.2]	0–63.7	11	388	3.4 [2.7–4.3]	91.5 [86.7–94.5]	< 0.001
Low risk of bias	38.3 [0–100]	NA	2	23	5.3 [3.2–8.7]	96.4 [90–98.7]	< 0.001
Process controlled	1.1 [0–9.4]	[0–48.5]	8	353	3.2 [2.4–4.2]	90 [82.8–94.2]	< 0.001
Surface water							
Overall	7.5 [2.4–14.5]	[0–49]	20	1271	3.5 [3–4.1]	92 [89–94.2]	< 0.001
Low risk of bias	2 [0.6–4.1]	[0–23.9]	3	329	1 [1–3.1]	0 [0–89.6]	0.448
Process controlled	8.5 [1.4–19.7]	[0–62.9]	11	804	4.3 [3.6–5.2]	94.6 [92.1–96.3]	< 0.001
Drinking water							
Overall	4.7 [0–15.9]	0–59.9	10	998	4.1 [3.3–5]	94 [90.9–96]	< 0.001
Process controlled	9.9 [0–42.9]	[0–100]	4	778	6.5 [5–8.5]	97.6 [95.9–98.6]	< 0.001
Groundwater							
Overall	8.3 [0–32.4]	NA	1	12	NA	NA	1
Others							
Overall	6 [0–33.2]	0–100	5	179	4.1 [3–5.6]	94.1 [89.1–96.8]	< 0.001
Low risk of bias	0 [0–7]	NA	1	24	NA	NA	1
Process controlled	0 [0–5.1]	NA	2	35	1 NA	0 NA	0.798

*95% CI* 95% confidence interval; *NA* not applicable

^¶^*H* is a measure of the extent of heterogeneity; a value of *H* > 1 indicates a potential heterogeneity of the prevalence of hepatitis E virus

^§^*I*^2^ describes the proportion of total variation in prevalence of hepatitis E virus that is due to heterogeneity; a value > 50% indicates the presence of heterogeneity

### Heterogeneity and Publication Bias

The degree of heterogeneity and publication bias within the prevalence data is presented in Table 1. The estimation of prevalence data was associated with significant heterogeneity (*H* > 1 and *I*^2^ > 50%) and the presence of publication bias (*p* < 0.05 for Egger’s test) in the different groups of water matrices. The publication bias results obtained by Egger’s test were confirmed by the funnel plot (Fig. S2).

### Subgroup Analyses

The subgroup analysis is presented in Table S5. The overall prevalence was significantly different according to countries (*p* < 0.001) with higher prevalence in Portugal (45.1%, 3 prevalence data), Pakistan (40.7%, 1 prevalence data), Germany (39.8%, 5 prevalence data), Sweden (35.8%, 2 prevalence data), and Switzerland (32.3%, 1 prevalence data). According to WHO region (*p* = 0.015), significantly higher prevalence was in Europe (12.2%, 56 prevalence data) and South-East Asia (9.9%, 10 prevalence data). For UNSD region (*p* = 0. 001), higher prevalence was in Western Europe (24.7%, 14 prevalence data), Northern Europe (17.6%, 6 prevalence data), and Southern Asia (14.3%, 10 prevalence data). Regarding country income level (*p* < 0.001), higher prevalence was in high-income economies (11.9%, 59 prevalence data).

Pooled prevalence did not differ significantly by sampling approach (*p* = 0.809), rural/urban setting (*p* = 0.059), type of water matrices (*p* = 0.307), and detection assays (*p* = 0.106).

## Discussion

Hepatitis E virus is a leading cause of waterborne viral hepatitis in most parts of Asia and Africa, causing outbreaks of variable magnitude, affecting several hundred to several thousand persons. In industrialized countries, instead, the disease is infrequent, and only occasional sporadic cases occur, due to zoonotic spread of genotype 3 or 4 HEV from animals, mainly through the consumption of undercooked meat (Ricci et al., 2017). A mathematical model to rank the importance of various types of food potentially implicated in the transmission of HEV to humans in the population has been recently published in Italy, suggesting that pork products with and without liver emerged as the most important food implicated in HEV transmission (Moro et al., 2021). It is not well known to what extent contaminated fruit, vegetables, bivalve molluscs contribute to transmission of HEV. Risk factors for sporadic hepatitis E infection have been recently investigated in a systematic review and meta-analysis (Pavio et al., 2021), highlighting that consumption of pork products and processed meat is highly at risk of HEV infection, but consumption of produce or shellfish or of other food products is also associated with HEV exposure; moreover, blood transfusion and dialysis are risk factors for HEV.

The control of HEV requires preventive measures, the main target of which is the transmission. Knowledge of different factors that may facilitate the transmission of HEV to humans is a necessity. The potential for waterborne transmission in industrialized countries has not been investigated even if occurrence of HEV has been documented in different water matrices.

This study aimed to assess the prevalence of HEV in water matrices. A total of 87 prevalence data were included, related to water samples collected over 30 years (1989–2019). The overall HEV prevalence in water matrices was 9.8%. Depending on the type of water matrix, the prevalence varied according to the degree of water pollution, with untreated wastewater showing, as expected, the higher prevalence (15.1%). Significant variability was observed between the different studies performed on untreated wastewater, with only five studies (performed in the United Kingdom, Egypt, Greece, Tunisia, Japan, and Sweden) showing the absence of HEV genome. Frequency of detection in the remaining 34 prevalence data varied between 1.32% in China to 93.33% in the United Kingdom. The vast majority of the data (*n* = 20) showed prevalence > 10%. The high prevalence in untreated water can be explained by the excretion of the virus via contaminated faeces or urine from infected individuals (Abravanel et al., 2015; Chandra et al., 2010; Geng et al., 2016; Goel et al., 2020; Takahashi et al., 2007). Before the onset of disease symptoms, up to 1 × 10^5^ HEV genome copies per gram of faeces can be excreted for several days (Li et al., 2006). Therefore, untreated wastewater can be used for community surveillance of hepatitis E, to investigate the circulation of the virus in the catchment area served by the wastewater treatment plant (WWTPs), as well to study viral diversity. Indeed, most of the studies included in the category untreated wastewater aimed at studying the epidemiology of HEV within a given population for the purpose of wastewater-based epidemiology.

The prevalence of HEV in treated wastewater (3.8%) was considerably lower than untreated wastewater. Indeed, HEV RNA was detected only in two of the 11 prevalence data, one from Germany (31.3%), and the other from Sweden (90.9%). No HEV RNA was detected in the remaining nine studies. This suggests that treatments applied at WWTPs are usually efficient in removing HEV. Indeed, several conventional methods are implemented in order to remove pathogens during wastewater treatment, including coagulation, filtration, chlorination, activated sludge treatment process, and anaerobic digestion (Nasir et al., 2022). Non-negligible prevalence (7.4%) was also found in surface water (river, lake, and sea). The higher prevalence (77.7%) was found in samples collected in Portugal, followed by Germany (30.0%), Italy, Philippines, and India (25% each), and the Netherlands (16.7%). Very low prevalence (range 3.2–7.7%) was found in the remaining studies. Surface pollution can be consequence of lack or improper wastewater treatment, resulting in wastewater effluent discharge into surface water sources (Okoh et al., 2010). Direct faecal contamination of the environment from humans and animals or run off from pig farm can also impact soil or surface waters, for example, by bathers or by defecation of wild animals (Rodríguez-Lázaro et al., 2012).

Of the ten studies related to drinking waters, four found HEV RNA, with prevalence ranging from 1.42% in India to 66.6% in Portugal. The latter study also found infectious HEV by cell cultures in 27.7% of the samples. The remaining studies did not report any detection of HEV RNA. Only one study has been published on HEV in groundwater, performed in Spain, resulting in one positive sample out of 12 analysed (8.33%). Finally, HEV was also detected in other water matrices, such effluents of a pig slaughterhouse (15/20 samples, 75%). HEV strains have been described from pigs worldwide, mostly belonging to genotypes 3 and 4 and sequences from autochthonous HEV in non-endemic countries have been demonstrated to be phylogenetically close to swine strains, demonstrating that zoonotic transmission of HEV is relevant in industrialized countries (Clemente-Casares et al., 2003). No HEV was detected in other water matrices such as grey water, flood water, irrigation water, and reclaimed water.

Overall, only 36.8% of the studies reported the genotype of HEV, with HEV-3 prevalent (168 samples), followed by HEV-1 (148 sample), and HEV-4 (2 samples).

In summary, several studies showed the occurrence of HEV in water matrices including those that came in direct contact with humans. However, it is important to note that RT-PCR and real-time PCR assays are able to detect only viral genome fragments, and do not provide information on viral infectivity. Thus, the presence of HEV genome in the different water matrices does not necessarily indicate an actual threat to human health.

It was also noted that the prevalence of HEV in water matrices varied greatly between regions of the world and countries, with high prevalence in Europe and North America. Despite this high prevalence of HEV in water matrices, the different methods of water treatment in these countries limit infectivity and transmission to humans, hence the lower seroprevalences of HEV in industrialised countries (Li et al., 2020).

The number of studies from low-income countries, as defined by the World Bank Data, was low (3/87, 3.5%, all from Uganda) as well as the total number of investigated samples, none of which showed the presence of HEV RNA. Seventeen studies were available from six lower–middle-income countries (Pakistan, Tunisia, Egypt, India, Philippines, and Kenia), showing HEV RNA in 9.63% of the samples, and five from upper–middle-income economies (Colombia, Brazil, China, Argentina, and Thailand), with prevalence of 3.1%. Finally, the vast majority of the studies were performed in 16 high-income countries (Italy, Germany, France, Spain, United States of America, United Kingdom, Greece, Switzerland, Portugal, Romania, Israel, The Netherland, Slovenia, Japan, Sweden, and New Zealand), with prevalence of 11.87%. The number of studies as well as the number of tested samples varied considerable among the four income groups of countries, therefore, we cannot draw conclusions based on differences linked to country income level. Moreover, high heterogeneity was documented in the selected studies in relation to methods used for sample collection (sample volume), viral concentration, RNA extraction, and target regions for HEV typing/subtyping. Less than 50% of the studies reported the use of a process control to monitor the efficiency of the virus concentration/extraction steps. This variability in the methods used makes it difficult to compare the results of the different studies.

This study shows that improving wastewater treatment and management is necessary to limit the contamination of surface water. This study also shows that contamination of drinking waters can also occur, questioning the quality of drinking water and wastewater treatment and highlighting the need for effective virological control of water for human consumption. A recent study performed in Sweden used next-generation sequencing to explore the virome in water from drinking water treatment plants and tap water and found that HEV passed through the treatment and entered into the supply network (Wang et al., 2020a). They suggested that the risk of getting infected through consumption of tap water is probably negligible, but needs to be investigated. Fenaux et al. also suggested the involvement of water matrices in HEV transmission in industrialized countries (Fenaux et al., 2019).

One of the limitations of this study is heterogeneity, since 62.4% of the studies were conducted in Europe, and almost half of the studies were conducted on one type of water matrix (untreated wastewater), while only one study was conducted on groundwater. Moreover, a significant heterogeneity in the techniques and detection methods was described.

Only 16.1% of the studies had a low risk of bias, which requires epidemiological studies of high methodological quality for future studies.

## Conclusion

This review found a not negligible prevalence of HEV in water matrices, especially untreated wastewater. With inadequate sewage treatment and poor hygiene, HEV can find its way to other water matrices and cause human diseases. However, given the lack of data on the infectivity of the virus in these matrices, no direct conclusion can be achieved on the risk associated with environmental contamination.

## Supplementary Information

Below is the link to the electronic supplementary material.

Supplementary file1 (DOCX 1462 kb)

## Data Availability

All data generated or analysed during this study are included in this published article [and its supplementary information files].
