# Supplementary material for: Hepatitis E Virus in Water Environments: A Systematic Review and Meta-analysis
Source: Food Environ Virol. 2022 Aug 29;14(3):223–35. doi: 10.1007/s12560-022-09530-3 (PMC9458591; doi:10.1007/s12560-022-09530-3)
Supplement: Supplementary file 1 — Supplementary file1 (DOCX 1462 kb) [file 12560_2022_9530_MOESM1_ESM.docx]

**Supplemental Material**

**Hepatitis E virus in water environments: a systematic review and meta-analysis**

Takuissu GR^1^, Kenmoe S^2^, Ndip L^2^, Ebogo-Belobo JT^3^, Kengne-Ndé C^4^, Mbaga DS^5^, Bowo-Ngandji A^5^, Oyono MG^6^, Kenfack-Momo R^7^, Tchatchouang S^8^, Kenfack-Zanguim J^7^, Lontuo Fogang R^9^, Zeuko'o Menkem E^10^, Kame-Ngasse GI^3^, Magoudjou-Pekam JN^7^, Nkie Esemu S^2^, Veneri C^11^, Mancini P^11^, Bonanno Ferraro G^11^, Iaconelli M^11^, Suffredini E^12^, La Rosa G^11^*.

1: Centre for Food, Food Security and Nutrition Research, Institute of Medical Research and Medicinal Plants Studies, Yaounde, Cameroon,

2: Department of Microbiology and Parasitology, University of Buea, Buea, Cameroon,

3: Medical Research Centre, Institute of Medical Research and Medicinal Plants Studies, Yaounde, Cameroon,

4: Epidemiological Surveillance, Evaluation and Research Unit, National AIDS Control Committee, Douala, Cameroon,

5: Department of Microbiology, The University of Yaounde I, Yaounde, Cameroon,

6: Centre for Research on Health and Priority Pathologies, Institute of Medical Research and Medicinal Plants Studies, Yaounde, Cameroon,

7: Department of Biochemistry, The University of Yaounde I, Yaounde, Cameroon,

8: Scientific Direction, Centre Pasteur du Cameroun, Yaounde, Cameroon,

9: Department of Animal Biology, University of Dschang, Dschang, Cameroon,

10: Department of Biomedical Sciences, University of Buea, Buea, Cameroon,

11: Department of Environment and Health, Istituto Superiore di Sanità, Rome, Italy

12: Department of Food Safety, Nutrition and Veterinary public health, Istituto Superiore di Sanità, Rome, Italy

*Corresponding Author: La Rosa G, Department of Environment and Health, Istituto Superiore di Sanità, Rome, Italy. [giuseppina.larosa@iss.it](mailto:giuseppina.larosa@iss.it)

**Table of Contents**

**Table S1:** Search strategy

**Table S2:** Items for risk of bias assessment

**Table S3:** Main reasons of exclusion of eligible studies

**Table S4:** Characteristics of included studies

**Table S5:** Detailed results of meta-analysis results for prevalence of hepatitis E virus in various water matrices.

**Fig S1:** Funnel chart for publications of the global hepatitis E virus prevalence in water matrices

**Table S1:** Search strategy

| **Search** | **Fields** |  |
| --- | --- | --- |
| #1 | HEV | Hepatitis E virus OR HEV OR Viral hepatitis E |
| #2 | Water environments | Water OR “Waste Water” OR Sewage OR Wastewater OR River OR “Surface water” OR Groundwater OR “brackish water” OR Seawater OR “sea water” OR “wastewater treatment plant” OR “drinking water” OR “tap water” OR “potable water” OR lake OR “fresh water” OR freshwater OR “marine water” |
| #3 |  | #1 AND #2 |

**Table S2:** Items for risk of bias assessment

| **Modified Hoy et al. tool for cross sectional studies** | **Yes (1)/No (0)** |
| --- | --- |
| ***External validity*** |  |
| 1. Was the study’s target population a close representation of the national population in relation to hepatitis E virus prevalence? | **1** |
| 2. Was the sampling frame a true or close representation of the samples? | **1** |
| 3. Was some form of random selection used to select the sample? | **1** |
| 4. Did the author calculate and respect the expected sample size? | **1** |
| ***Internal validity*** |  |
| 5. Was an acceptable water matrix definition used in the study? | **1** |
| 6. Was the HEV detection assay shown to have validity and reliability? | **1** |
| 7. Was the same mode of data collection used for all samples? | **1** |
| 8. Was the length of the study period > or = 1 year? | **1** |
| 9. Were the numerator(s) and denominator(s) for the prevalence of hepatitis E virus reported? | **1** |
| ***Total score*** | **9** |
| **Interpretation of the risk of bias tool**   - 7-9: Low risk of bias - 4-6: Moderate risk of bias - 0-3: High risk of bias |  |

*Modified from:*  Hoy D, Brooks P, Woolf A, Blyth F, March L, Bain C, et al. Assessing risk of bias in prevalence studies: modification of an existing tool and evidence of interrater agreement. J Clin Epidemiol. 2012;65: 934–939. doi:10.1016/j.jclinepi.2011.11.014

**Table S3:** Main reasons of exclusion of eligible studies

| N° | Author, Year | Title | Reason of exclusion |
| --- | --- | --- | --- |
| 1 | Abe, 2006 | International collaborative survey on epidemiology of hepatitis E virus in 11 countries. | No data on HEV prevalence in wastewater |
| 2 | Acharya, 2011 | Hepatitis E: water, water everywhere - now a global disease. | Review |
| 3 | Adriaenssens, 2018 | Viromic Analysis of Wastewater Input to a River Catchment Reveals a Diverse Assemblage of RNA Viruses. | No data on HEV prevalence in wastewater |
| 4 | Aggarwal, 1994 | Hepatitis E: Intrafamilial transmission versus waterborne spread. | No data on HEV prevalence in wastewater |
| 5 | Aggarwal, 2000 | Hepatitis E and intrafamilial transmission. | No data on HEV prevalence in wastewater |
| 6 | Aguado, 2019 | VirWaTest, A Point-of-Use Method for the Detection of Viruses in Water Samples. | No data on HEV prevalence in wastewater |
| 7 | Ahmad, 2015 | Frequency of hepatitis E and Hepatitis A virus in water sample collected from Faisalabad, Pakistan. | No data on HEV prevalence in wastewater |
| 8 | Al Mahtab, 2013 | Epidemiology of hepatitis E virus in an urban community in Dhaka city. | No data on HEV prevalence in wastewater |
| 9 | Al-Batanony, 2011 | Work-related health effects among wastewater treatment plants workers. | No data on HEV prevalence in wastewater |
| 10 | Albinana-Gimenez, 2006 | Distribution of human polyomaviruses, adenoviruses, and hepatitis E virus in the environment and in a drinking-water treatment plant. | No data on HEV prevalence in wastewater |
| 11 | Ali, 2004 | Detection of enteric viruses, Giardia and Cryptosporidium in two different types of drinking water treatment facilities. | No data on HEV prevalence in wastewater |
| 12 | Al-Nasrawi, 2010 | Viral hepatitis e outbreak in Al-Sadr city, Baghdad, Iraq. | No data on HEV prevalence in wastewater |
| 13 | Anastasi, 2008 | Virus removal in conventional wastewater treatment process. | No data on HEV prevalence in wastewater |
| 14 | Apostol, 2012 | Genetic diversity and molecular characterization of enteroviruses from sewage-Polluted urban and rural rivers in the Philippines. | No data on HEV prevalence in wastewater |
| 15 | Arankalle, 2001 | Changing epidemiology of hepatitis A and hepatitis E in urban and rural India (1982-98). | No data on HEV prevalence in wastewater |
| 16 | Arankalle, 1994 | Seroepidemiology of water-borne hepatitis in India and evidence for a third enterically-transmitted hepatitis agent. | No data on HEV prevalence in wastewater |
| 17 | Archer, 1954 | An epidemic of infective hepatitis apparently due to a water-borne agent. | No data on HEV prevalence in wastewater |
| 18 | Arif, 1994 | Epidemiology of hepatitis E virus (HEV) infection in Saudi Arabia. | No data on HEV prevalence in wastewater |
| 19 | Arnone, 2007 | Waterborne pathogens in urban watersheds. | No data on HEV prevalence in wastewater |
| 20 | Arora, 2013 | Water borne hepatitis A and hepatitis E in Malwa Region of Punjab, India. | No data on HEV prevalence in wastewater |
| 21 | Ashbolt, 2004 | Microbial contamination of drinking water and disease outcomes in developing regions. | Review |
| 22 | Awsathi, 2014 | Epidemiological investigation of the jaundice outbreak in lalkuan, nainital district, uttarakhand. | No data on HEV prevalence in wastewater |
| 23 | Bae, 2022 | Development of diagnostic systems for wide range and highly sensitive detection of two waterborne hepatitis viruses from groundwater using the conventional reverse transcription nested PCR assay. | No data on HEV prevalence in wastewater |
| 24 | Bagulo, 2022 | Hepatitis E seroprevalence and risk factors in humans and pig in Ghana. | No data on HEV prevalence in wastewater |
| 25 | Baicus, 2016 | Short communication: Could the genexpert system be a new tool for poliovirus detection in the sewage water? | Case report |
| 26 | Banerjee, 2005 | Outbreak of viral hepatitis E in a regimental training centre. | No data on HEV prevalence in wastewater |
| 27 | Baylis, 2010 | Widespread distribution of hepatitis E virus in plasma fractionation pools. | No data on HEV prevalence in wastewater |
| 28 | Belabbes, 1983 | Epidemic non-A, non-B viral hepatitis in Algeria: Strong evidence for its spreading by water. | No data on HEV prevalence in wastewater |
| 29 | Benckert, 2011 | [Diagnosis and clinical features of infection with hepatitis A and hepatitis E viruses. Transmission through drinking water and foodstuffs]. | Review |
| 30 | Bile, 1994 | Contrasting roles of rivers and wells as sources of drinking water on attack and fatality rates in a hepatitis E epidemic in Somalia. | Case report |
| 31 | Bosch, 1995 | The survival of enteric viruses in the water environment. | Full text or abstract not found |
| 32 | Bosch, 2011 | Analytical Methods for Virus Detection in Water and Food. | No data on HEV prevalence in wastewater |
| 33 | Botzenhart, 2007 | Viruses in drinking water. | Article not in English or in French |
| 34 | Brassard, 2011 | Simultaneous recovery of bacteria and viruses from contaminated water and spinach by a filtration method. | No data on HEV prevalence in wastewater |
| 35 | Brisebois, 2018 | Human viral pathogens are pervasive in wastewater treatment center aerosols. | No data on HEV prevalence in wastewater |
| 36 | Buisson, 1993 | Water and viral hepatitis. | No data on HEV prevalence in wastewater |
| 37 | Buti, 2004 | Sporadic cases of acute autochthonous hepatitis E in Spain. | No data on HEV prevalence in wastewater |
| 38 | Cabezas Sánchez, 2018 | Infectious diseases related to water in Peru. | Review |
| 39 | Calgua, 2013 | New methods for the concentration of viruses from urban sewage using quantitative PCR. | No data on HEV prevalence in wastewater |
| 40 | Carducci, 2008 | Study of the viral removal efficiency in a urban wastewater treatment plant. | No data on HEV prevalence in wastewater |
| 41 | Carducci, 2006 | Epidemiological surveillance of human enteric viruses by monitoring of differnet environmental matrices. | No data on HEV prevalence in wastewater |
| 42 | Carratalà, 2019 | Population density and water balance influence the global occurrence of hepatitis E epidemics. | Review |
| 43 | Ceylan, 2003 | A special risk group for hepatitis E infection: Turkish agricultural workers who use untreated waste water for irrigation. | No data on HEV prevalence in wastewater |
| 44 | Chacón, 2011 | Wastewater-based epidemiology of enteric viruses and surveillance of acute gastrointestinal illness outbreaks in a resource-limited region. | No data on HEV prevalence in wastewater |
| 45 | Chang, 1960 | Survival, and protection against chlorination, of human enteric pathogens in free-living nematodes isolated from water supplies. | No data on HEV prevalence in wastewater |
| 46 | Chen, 2016 | Epidemiological investigation of a tap water-mediated hepatitis E virus genotype 4 outbreak in Zhejiang Province, China. | Sample size < or = 10 participants |
| 47 | Corwin, 1995 | Two years' investigation of epidemic hepatitis E virus transmission in West Kalimantan (Borneo), Indonesia. | No data on HEV prevalence in wastewater |
| 48 | Corwin, 1997 | Epidemic and sporadic hepatitis E virus transmission in West Kalimantan (Borneo), Indonesia. | No data on HEV prevalence in wastewater |
| 49 | Corwin, 1996 | A waterborne outbreak of hepatitis E virus transmission in southwestern Vietnam. | No data on HEV prevalence in wastewater |
| 50 | Corwin, 1999 | The unique riverine ecology of hepatitis E virus transmission in South-East Asia. | No data on HEV prevalence in wastewater |
| 51 | Courault, 2017 | Assessment and risk modeling of airborne enteric viruses emitted from wastewater reused for irrigation. | No data on HEV prevalence in wastewater |
| 52 | Crossan, 2012 | Hepatitis E Virus Genotype 3 in Shellfish, United Kingdom. | No data on HEV prevalence in wastewater |
| 53 | Cuevas-Ferrando, 2020 | Assessment of ISO Method 15216 to Quantify Hepatitis E Virus in Bottled Water. | No data on HEV prevalence in wastewater |
| 54 | Cummings, 2014 | Hepatitis E in Karamoja, Uganda, 2009-2012: Epidemiology and challenges to control in a setting of semi-nomadic pastoralism. | No data on HEV prevalence in wastewater |
| 55 | Dalton, 2018 | Transmission and epidemiology of hepatitis e virus genotype 3 and 4 infections. | No data on HEV prevalence in wastewater |
| 56 | de Lambert, 2021 | Microbial pathogens and contaminants of emerging concern in groundwater at an urban subsurface stormwater infiltration site. | No data on HEV prevalence in wastewater |
| 57 | De Melo Cassemiro, 2016 | Molecular and phenotypic characterization of a highly evolved type 2 vaccine-derived poliovirus isolated from seawater in Brazil, 2014. | No data on HEV prevalence in wastewater |
| 58 | De Wilde, 1998 | Hepatitis E. | No data on HEV prevalence in wastewater |
| 59 | Denis-Mize, 2004 | Detection of human enteric viruses in stream water with RT-PCR and cell culture. | No data on HEV prevalence in wastewater |
| 60 | dos Santos, 2011 | Hepatitis E virus in swine and effluent samples from slaughterhouses in Brazil | Sample size < or = 10 participants |
| 61 | D'Ugo, 2016 | Detection of Human Enteric Viruses in Freshwater from European Countries. | No data on HEV prevalence in wastewater |
| 62 | Dwibedi, 2018 | An outbreak of hepatitis E virus infection caused by genotype 1 in an urban setting in eastern India: a probe into risk factors for transmission. | No data on HEV prevalence in wastewater |
| 63 | Elmahdy, 2016 | Spatial distribution of enteric viruses and somatic coliphages in a Lagoon used as drinking water source and recreation in Southern Brazil. | No data on HEV prevalence in wastewater |
| 64 | Emerson, 2004 | Global health - Running like water - The omnipresence of hepatitis E. | No data on HEV prevalence in wastewater |
| 65 | Fabres, 2017 | Evaluation of virus recovery methods and efficiency of tannin-derived coagulants in removing total coliforms, E-coli and enteric viruses in effluents of a domestic sewage treatment plant. | No data on HEV prevalence in wastewater |
| 66 | Faleye, 2021 | Wastewater-based epidemiology and long-read sequencing to identify enterovirus circulation in three municipalities in maricopa county, arizona, southwest united states between june and october 2020. | No data on HEV prevalence in wastewater |
| 67 | Fenaux, 2019 | Transmission of hepatitis E virus by water: An issue still pending in industrialized countries. | No data on HEV prevalence in wastewater |
| 68 | Fernández-Barredo, 2006 | Detection of hepatitis E virus shedding in feces of pigs at different stages of production using reverse transcription-polymerase chain reaction. | No data on HEV prevalence in wastewater |
| 69 | Fernandez-Cassi, 2018 | Metagenomics for the study of viruses in urban sewage as a tool for public health surveillance. | No data on HEV prevalence in wastewater |
| 70 | Fongaro, 2015 | Human and animal enteric virus in groundwater from deep wells, and recreational and network water. | No data on HEV prevalence in wastewater |
| 71 | Fowotade, 2018 | Seroprevalence of hepatitis E among restaurant food handlers in Ibadan, Nigeria. | No data on HEV prevalence in wastewater |
| 72 | Froese, 1999 | Health effects associated with wastewater treatment, disposal, and reuse. | No data on HEV prevalence in wastewater |
| 73 | Fusco, 2019 | Detection of Hepatitis A Virus and Other Enteric Viruses in Shellfish Collected in the Gulf of Naples, Italy. | No data on HEV prevalence in wastewater |
| 74 | Gao, 2016 | Development and evaluation of a RT-LAMP assay for rapid detection of hepatitis E virus from shellfish. | No data on HEV prevalence in wastewater |
| 75 | Gao, 2015 | Surveillance of hepatitis e virus contamination in shellfish in china. | No data on HEV prevalence in wastewater |
| 76 | Geng, 2016 | Transmission of hepatitis e virus. | Review |
| 77 | Gerba, 1988 | Enterovirus detection in water with gene probes. | No data on HEV prevalence in wastewater |
| 78 | Ghannoum, 1981 | The incidence of water-related diseases in the Brak area, Libya from 1977 to 1979, before and after the installation of water treatment plants. | No data on HEV prevalence in wastewater |
| 79 | Gibson, 2011 | Evaluation of human enteric viruses in surface water and drinking water resources in southern Ghana. | Sample size < or = 10 participants |
| 80 | Givens, 2016 | Detection of hepatitis E virus and other livestock-related pathogens in Iowa streams. | No data on HEV prevalence in wastewater |
| 81 | Giwa, 2020 | Dissecting microbial community structure in sewage treatment plant for pathogens’ detection using metagenomic sequencing technology. | No data on HEV prevalence in wastewater |
| 82 | Gomes, 2019 | Removal of Enteric Pathogens from Real Wastewater Using Single and Catalytic Ozonation. | No data on HEV prevalence in wastewater |
| 83 | Grabow, 1997 | Hepatitis viruses in water: Update on risk and control. | Full text or abstract not found |
| 84 | Grimm, 2002 | Development of a molecular method to identify hepatitis E virus in water. | No data on HEV prevalence in wastewater |
| 85 | Guerrero-Latorre, 2011 | Occurrence of water-borne enteric viruses in two settlements based in Eastern Chad: Analysis of hepatitis E virus, hepatitis A virus and human adenovirus in water sources. | Sample size < or = 10 participants |
| 86 | Guerrero-Latorre, 2016 | UV disinfection and flocculation-chlorination sachets to reduce hepatitis E virus in drinking water. | No data on HEV prevalence in wastewater |
| 87 | Guerrero-Latorre, 2016 | Transmission Sources of Waterborne Viruses in South Sudan Refugee Camps. | Sample size < or = 10 participants |
| 88 | Guerrero-Latorre, 2018 | Quito's virome: Metagenomic analysis of viral diversity in urban streams of Ecuador's capital city. | No data on HEV prevalence in wastewater |
| 89 | Guthmann, 2006 | A large outbreak of hepatitis E among a displaced population in Darfur, Sudan, 2004: The role of water treatment methods. | No data on HEV prevalence in wastewater |
| 90 | Hamza, 2017 | Detection and characterization of hepatitis A virus circulating in Egypt. | No data on HEV prevalence in wastewater |
| 91 | Harnagle, 2014 | An epidemiological investigation of an outbreak of viral hepatitis E at Ahmednagar, Maharashtra, India. | No data on HEV prevalence in wastewater |
| 92 | Hartard, 2021 | Variability in molecular characteristics of Hepatitis E virus quasispecies could modify viral surface properties and transmission. | No data on HEV prevalence in wastewater |
| 93 | Hau, 1999 | Prevalence of enteric hepatitis A and E viruses in the Mekong River delta region of Vietnam. | No data on HEV prevalence in wastewater |
| 94 | Hellmér, 2014 | Detection of pathogenic viruses in sewage provided early warnings of hepatitis A virus and norovirus outbreaks. | No data on HEV prevalence in wastewater |
| 95 | Hennechart-Collette, 2021 | Detection of norovirus, hepatitis A and hepatitis E viruses in multicomponent foodstuffs. | No data on HEV prevalence in wastewater |
| 96 | Hrdy, 2021 | MOL-PCR and xMAP Technology: A Multiplex System for Fast Detection of Food- and Waterborne Viruses. | No data on HEV prevalence in wastewater |
| 97 | Hunter, 2012 | Hepatitis E (HEV) in South West England. Geographical, environmental and social factors: A case control study. | No data on HEV prevalence in wastewater |
| 98 | Ianiro, 2021 | Long-term surveillance for hepatitis E virus in an Italian two-site farrow-to-finish swine farm. | No data on HEV prevalence in wastewater |
| 99 | Iarasheva, 1993 | The epidemiological diagnosis of "fecal-oral" hepatitis E in Tajikistan. | No data on HEV prevalence in wastewater |
| 100 | Irshad, 1999 | Hepatitis E virus: An update on its molecular, clinical and epidemiological characteristics. | No data on HEV prevalence in wastewater |
| 101 | Isaäcson, 2000 | An outbreak of hepatitis E in Northern Namibia, 1983. | No data on HEV prevalence in wastewater |
| 102 | Ishida, 2012 | Detection and molecular characterization of hepatitis E virus in clinical, environmental and putative animal sources. | No data on HEV prevalence in wastewater |
| 103 | Ishii , 2014 | Microfluidic quantitative PCR for simultaneous quantification of multiple viruses in environmental water samples. | No data on HEV prevalence in wastewater |
| 104 | Ivanova, 1986 | Isolation of enteroviruses from water. | No data on HEV prevalence in wastewater |
| 105 | Jeggli, 2003 | Hepatitis E and exposure to waste water. | No data on HEV prevalence in wastewater |
| 106 | Jothikumar, 1993 | Detection of hepatitis E virus in raw and treated wastewater with the polymerase chain reaction. | No data on HEV prevalence in wastewater |
| 107 | Junaid , 2014 | Sanitary Survey of Drinking Water Quality in Plateau State, Nigeria. | No data on HEV prevalence in wastewater |
| 108 | Jung, 2011 | Occurrence of norovirus and other enteric viruses in untreated groundwaters of Korea. | No data on HEV prevalence in wastewater |
| 109 | Kaas, 2019 | Detection of Human Enteric Viruses in French Polynesian Wastewaters, Environmental Waters and Giant Clams. | Sample size < or = 10 participants |
| 110 | Kadri, 2018 | Hepatitis A and E Outbreak Surveillance during 2015-2017 in Kashmir, India: Is the Water to Blame? | No data on HEV prevalence in wastewater |
| 111 | Kamar, 2017 | Hepatitis E virus infection. | No data on HEV prevalence in wastewater |
| 112 | Kase, 2009 | Detection and molecular characterization of swine hepatitis E virus in North Carolina swine herds and their faecal wastes. | No data on HEV prevalence in wastewater |
| 113 | Kasorndorkbua, 2005 | Infectious swine hepatitis E virus is present in pig manure storage facilities on United States farms, but evidence of water contamination is lacking. | No data on HEV prevalence in wastewater |
| 114 | Kitajima, 2009 | First detection of genotype 3 hepatitis E virus RNA in river water in Cambodia. | No data on HEV prevalence in wastewater |
| 115 | Kluge, 2014 | Human adenovirus (HAdV), human enterovirus (hEV), and genogroup A rotavirus (GARV) in tap water in southern Brazil. | No data on HEV prevalence in wastewater |
| 116 | Kokkinos, 2017 | Virological Quality of Irrigation Water in Leafy Green Vegetables and Berry Fruits Production Chains. | No data on HEV prevalence in wastewater |
| 117 | Kokkinos, 2011 | Molecular detection of multiple viral targets in untreated urban sewage from Greece. | Duplicates |
| 118 | Kollins, 1966 | The presence of human enteric viruses in sewage and their removal by conventional sewage treatment methods. | Review |
| 119 | Krasilnikov, 1985 | Isolation of viruses from water using porous silica. | Article not in English or in French |
| 120 | Kretschmer, 1972 | Water and sewage as transmission factors in viral hepatitis, demonstrated by practical examples. | Article not in English or in French |
| 121 | Kumar, 2002 | Parenteral transmission of hepatitis E virus? Inferring too much too soon! | Full text or abstract not found |
| 122 | Kumar, 2019 | Jaundice outbreak likely caused by HEV in Amritsar, Punjab, India, 2013. | No data on HEV prevalence in wastewater |
| 123 | Kuroda, 2015 | Pepper mild mottle virus as an indicator and a tracer of fecal pollution in water environments: Comparative evaluation with wastewater-tracer pharmaceuticals in Hanoi, Vietnam. | No data on HEV prevalence in wastewater |
| 124 | Lenglet, 2020 | Does community-wide water chlorination reduce hepatitis E virus infections during an outbreak? A geospatial analysis of data from an outbreak in Am Timan, Chad (2016–2017). | No data on HEV prevalence in wastewater |
| 125 | Li, 2007 | Short report: Detection of hepatitis E virus RNA from the bivalve Yamato-Shijimi (Corbicula japonica) in Japan. | No data on HEV prevalence in wastewater |
| 126 | Li, 2017 | [Genotype distribution of human enteroviruses isolated from swage in Shanghai during year 2013-2014]. | No data on HEV prevalence in wastewater |
| 127 | Lipp, 2007 | Analysis of multiple enteric viral targets as sewage markers in coral reefs. | No data on HEV prevalence in wastewater |
| 128 | Liu, 2016 | Identification of Distribution Characteristics and Epidemic Trends of Hepatitis E in Zhejiang Province, China from 2007 to 2012. | No data on HEV prevalence in wastewater |
| 129 | Locas, 2010 | Removal of human enteric viruses and indicator microorganisms from domestic wastewater by aerated lagoons. | No data on HEV prevalence in wastewater |
| 130 | López-Gálvez, 2016 | Occurrence of enteric viruses in reclaimed and surface irrigation water: relationship with microbiological and physicochemical indicators. | No data on HEV prevalence in wastewater |
| 131 | Lund, 1979 | The survival of viral pathogens in water and waste in the tropics. | Full text or abstract not found |
| 132 | Mahnel, 1977 | Stability in drinking and surface water of nine virus species from different genera (author's transl). | No data on HEV prevalence in wastewater |
| 133 | Maila, 2004 | Identification of a new strain of hepatitis E virus from an outbreak in Namibia in 1995. | No data on HEV prevalence in wastewater |
| 134 | Martin-Latil, 2012 | Duplex RT-qPCR for the detection of hepatitis E virus in water, using a process control. | No data on HEV prevalence in wastewater |
| 135 | Martin-Latil, 2014 | Method for HEV detection in raw pig liver products and its implementation for naturally contaminated food. | No data on HEV prevalence in wastewater |
| 136 | Martolia, 2009 | An outbreak of hepatitis E tracked to a spring in the foothills of the Himalayas, India, 2005. | No data on HEV prevalence in wastewater |
| 137 | Masciopinto, 2019 | Human health risk assessment for the occurrence of enteric viruses in drinking water from wells: Role of flood runoff injections. | No data on HEV prevalence in wastewater |
| 138 | Masclaux, 2014 | Assessment of airborne virus contamination in wastewater treatment plants. | No data on HEV prevalence in wastewater |
| 139 | Matos, 2016 | Hepatitis e virus subgenotypes 3i and 3f in wastewater of treatment plants of Portugal. | Duplicates |
| 140 | McCall, 2021 | Assessment of enteric viruses during a hepatitis outbreak in Detroit MI using wastewater surveillance and metagenomic analysis. | No data on HEV prevalence in wastewater |
| 141 | Meng, 2011 | From barnyard to food table: The omnipresence of hepatitis E virus and risk for zoonotic infection and food safety. | Review |
| 142 | Metcalf, 1995 | Environmental virology: From detection of virus in sewage and water by isolation to identification by molecular biology - A trip of over 50 years. | Review |
| 143 | Mikhailov, 2013 | Possible water-borne HEV infection in a non-hyperendemic region. | No data on HEV prevalence in wastewater |
| 144 | Molinié, 1996 | Viral hepatitis E. | Review |
| 145 | Moore, 1993 | Surveillance for waterborne disease outbreaks--United States, 1991-1992. | Case report |
| 146 | Mosley, 1959 | Water-borne infectious hepatitis. | Review |
| 147 | Mosley, 1957 | Infectious hepatitis; report of an outbreak probably caused by drinking water. | Case report |
| 148 | Myremel, 2006 | Enteric viruses in inlet and outlet samples from sewage treatment plants. | No data on HEV prevalence in wastewater |
| 149 | Naik, 1992 | A large waterborne viral hepatitis E epidemic in Kanpur, India. | No data on HEV prevalence in wastewater |
| 150 | Naoumov, 2007 | Hepatitis A and E. | Review |
| 151 | Nasrullah, 2001 | <An> outbreak of viral hepatitis E. | No data on HEV prevalence in wastewater |
| 152 | Ndiaye, 2014 | Environmental surveillance of poliovirus and non-polio enterovirus in urban sewage in Dakar, Senegal (2007-2013). | No data on HEV prevalence in wastewater |
| 153 | Nedachin, 2015 | WASTE WATERS AS THE RESERVOIR OF INTESTINAL ENTERIC VIRAL INFECTIONS. | No data on HEV prevalence in wastewater |
| 154 | No author listed, 2004 | Hepatitis E fact sheet (revised August 2004). | Review |
| 155 | No author listed, 2004 | Hepatitis E, Chad. | No data on HEV prevalence in wastewater |
| 156 | No author listed, 2011 | Establishment of a viral hepatitis surveillance system--Pakistan, 2009-2011. | No data on HEV prevalence in wastewater |
| 157 | Noble, 2001 | Enteroviruses detected by reverse transcriptase polymerase chain reaction from the coastal waters of Santa Monica Bay, California: low correlation to bacterial indicator levels. | No data on HEV prevalence in wastewater |
| 158 | O'Brien, 2017 | Viral diversity and abundance in polluted waters in Kampala, Uganda. | No data on HEV prevalence in wastewater |
| 159 | Orduña, 1992 | [Hepatitis E virus]. | No data on HEV prevalence in wastewater |
| 160 | Oshiki, 2018 | Microfluidic PCR amplification and MiSeq amplicon sequencing techniques for high-throughput detection and genotyping of human pathogenic RNA viruses in human feces, sewage, and oysters. | No data on HEV prevalence in wastewater |
| 161 | Osuolale, 2017 | Human enteric bacteria and viruses in five wastewater treatment plants in the Eastern Cape, South Africa. | No data on HEV prevalence in wastewater |
| 162 | Parashar , 2011 | Survival of hepatitis A and E viruses in soil samples. | No data on HEV prevalence in wastewater |
| 163 | Park, 2010 | Human enteric viruses in groundwater. | No data on HEV prevalence in wastewater |
| 164 | Pawar, 2019 | A virus precipitation method for concentration & detection of avian influenza viruses from environmental water resources & its possible application in outbreak investigations. | No data on HEV prevalence in wastewater |
| 165 | Peczenick, 1956 | An apparently water-borne outbreak of infectious hepatitis. | No data on HEV prevalence in wastewater |
| 166 | Petrinca, 2009 | Presence and environmental circulation of enteric viruses in three different wastewater treatment plants. | No data on HEV prevalence in wastewater |
| 167 | Phanuwan, 2006 | Monitoring of human enteric viruses and coliform bacteria in waters after urban flood in Jakarta, Indonesia. | No data on HEV prevalence in wastewater |
| 168 | Pilevar, 2021 | Recent advances in biosensors for detecting viruses in water and wastewater. | Review |
| 169 | Pischke, 2013 | Hepatitis e virus infection: Multiple faces of an underestimated problem. | Review |
| 170 | Pitkanen, 2011 | Microbial Contamination of Groundwater at Small Community Water Supplies in Finland. | No data on HEV prevalence in wastewater |
| 171 | Poskanzer, 1961 | Waterborne infectious hepatitis epidemic Orom a chlorinated municipal supply. | No data on HEV prevalence in wastewater |
| 172 | Prado, 2014 | Detection of enteric viruses in activated sludge by feasible concentration methods. | No data on HEV prevalence in wastewater |
| 173 | Primavesi, 1965 | Results of virological investigations of sewage. | Full text or abstract not found |
| 174 | Puntaric, 1995 | Human virus detection in drinking water in Zagreb from 1991 to 1994. | Full text or abstract not found |
| 175 | Purpari, 2018 | Detection of human enteric viruses from shellfish, vegetable and water samples collected in Sicily. | No data on HEV prevalence in wastewater |
| 176 | Pusch, 2005 | Detection of enteric viruses and bacterial indicators in German environmental waters. | No data on HEV prevalence in wastewater |
| 177 | Rab, 1997 | Water-borne hepatitis E virus epidemic in Islamabad, Pakistan: A common source outbreak traced to the malfunction of a modern water treatment plant. | No data on HEV prevalence in wastewater |
| 178 | Ranjan, 2017 | Clinical Profile and Outcome of Pregnant Patients with Acute HEV Hepatitis During Water Borne Epidemic in Himachal Pradesh: A Hospital Based Study. | No data on HEV prevalence in wastewater |
| 179 | Rashid, 2021 | Detection of Human Adenovirus, Rotavirus, and Enterovirus in Tap Water and Their Association with the Overall Quality of Water in Karachi, Pakistan. | No data on HEV prevalence in wastewater |
| 180 | Rawat, 2016 | Viral Hepatitis A and E Outbreaks in Kumaon Region of Uttarakhand. | No data on HEV prevalence in wastewater |
| 181 | Rey, 2002 | Sero-epidemiological survey of hepatitis A and E epidemic in Mitrovica, Kosovo (1999). | No data on HEV prevalence in wastewater |
| 182 | Rodríguez-Lázaro, 2012 | Virus hazards from food, water and other contaminated environments. | No data on HEV prevalence in wastewater |
| 183 | Rodriguez-Manzano, 2010 | Analysis of the evolution in the circulation of HAV and HEV in Eastern Spain by testing urban sewage samples. | No data on HEV prevalence in wastewater |
| 184 | Sailaja, 2009 | Outbreak of waterborne hepatitis E in Hyderabad, India, 2005. | No data on HEV prevalence in wastewater |
| 185 | Salam, 2021 | Assessing the drinking water quality of educational institutions at selected locations of district Swat, Pakistan. | No data on HEV prevalence in wastewater |
| 186 | Santos-Ferreira, 2020 | Hepatitis E virus genotype 3 in echinoderms: First report of sea urchin (Paracentrotus lividus) contamination. | No data on HEV prevalence in wastewater |
| 187 | Sayed, 2020 | Circulation of hepatitis E virus (HEV) and/or HEV-like agent in non-mixed dairy farms could represent a potential source of infection for Egyptian people. | No data on HEV prevalence in wastewater |
| 188 | Schlindwein, 2010 | Detection of enteric viruses in sewage sludge and treated wastewater effluent. | No data on HEV prevalence in wastewater |
| 189 | Schvoerer, 2000 | PCR detection of human enteric viruses in bathing areas, waste waters and human stools in southwestern France. | No data on HEV prevalence in wastewater |
| 190 | Scipioni, 2000 | Contamination of food and water by human pathogenic viruses. | No data on HEV prevalence in wastewater |
| 191 | Sedyaningsih-Mamahit, 2002 | First documented outbreak of hepatitis E virus transmission in Java, Indonesia. | No data on HEV prevalence in wastewater |
| 192 | Seo, 2014 | Seasonal and regional prevalence of norovirus, hepatitis A virus, hepatitis E virus, and rotavirus in shellfish harvested from South Korea. | No data on HEV prevalence in wastewater |
| 193 | Singh, 2006 | Epidemiological investigation of an outbreak of viral hepatitis. | No data on HEV prevalence in wastewater |
| 194 | Singh, 1998 | Routes of transmission in the hepatitis E epidemic of Saharanpur. | No data on HEV prevalence in wastewater |
| 195 | Sinha, 2019 | Waterborne & foodborne viral hepatitis: A public health perspective. | No data on HEV prevalence in wastewater |
| 196 | Siniak, 1994 | Experience in verifying hepatitis E in Ukraine. | Article not in English or in French |
| 197 | Skidmore, 1992 | Hepatitis E virus: The cause of a waterbourne hepatitis outbreak. | No data on HEV prevalence in wastewater |
| 198 | Snowdon, 1989 | Coliphages as indicators of human enteric viruses in groundwater. | Review |
| 199 | Solodovnikov, 1995 | Viral hepatitis E: the epidemiological role of the water factor seen retrospectively and prospectively. | Article not in English or in French |
| 200 | Sorensen, 2021 | Seasonality of enteric viruses in groundwater-derived public water sources. | Not possible to extract data on HEV prevalence |
| 201 | Spina, 2018 | Learning from water treatment and hygiene interventions in response to a hepatitis E outbreak in an open setting in Chad. | No data on HEV prevalence in wastewater |
| 202 | Sree Kalpana, 2016 | Hepatitis E outbreak among factory workers due to contaminated factory water, Mandya District, Karnataka, India, 2015. | No data on HEV prevalence in wastewater |
| 203 | Sreenivasan, 1984 | A sero-epidemiologic study of a water-borne epidemic of viral hepatitis in Kolhapur City, India. | No data on HEV prevalence in wastewater |
| 204 | Swain, 2010 | A hepatitis E outbreak caused by a temporary interruption in a municipal water treatment system, Baripada, Orissa, India, 2004. | No data on HEV prevalence in wastewater |
| 205 | Symonds, 2009 | Eukaryotic viruses in wastewater samples from the United States. | No data on HEV prevalence in wastewater |
| 206 | Teixeira, 2020 | Quantitative PCR Detection of Enteric Viruses in Wastewater and Environmental Water Sources by the Lisbon Municipality: A Case Study. | No data on HEV prevalence in wastewater |
| 207 | Toole, 2006 | Hepatitis E virus infection as a marker for contaminated community drinking water sources in Tibetan villages. | No data on HEV prevalence in wastewater |
| 208 | Tripathy, 2019 | Study of a hepatitis e virus outbreak involving drinking water and sewage contamination in Shimla, India, 2015-2016. | Sample size < or = 10 participants |
| 209 | Tripathy, 2021 | An outbreak of hepatitis E in Yavatmal, India, 2019. | Sample size < or = 10 participants |
| 210 | Trmal, 2013 | Differences in the incidence of viral hepatitis A and E in the Czech Republic. | Article not in English or in French |
| 211 | Tschopp, 2009 | Hepatitis E, Helicobacter pylori and peptic ulcers in workers exposed to sewage: A prospective cohort study. | No data on HEV prevalence in wastewater |
| 212 | Tucker, 1954 | An outbreak of infectious hepatitis apparently transmitted through water. | No data on HEV prevalence in wastewater |
| 213 | Upfold, 2021 | Occurrence of Human Enteric Viruses in Water Sources and Shellfish: A Focus on Africa. | Review |
| 214 | Vaidya, 2003 | Increased Risk of Hepatitis E in Sewage Workers from India. | No data on HEV prevalence in wastewater |
| 215 | Van Zyl, 2019 | Detection of potentially pathogenic enteric viruses in environmental samples from Kenya using the bag-mediated filtration system. | No data on HEV prevalence in wastewater |
| 216 | Vecchia, 2012 | Assessment of enteric viruses in a sewage treatment plant located in Porto Alegre, southern Brazil. | No data on HEV prevalence in wastewater |
| 217 | Villar, 2007 | Molecular detection of hepatitis A virus in urban sewage in Rio de Janeiro, Brazil. | No data on HEV prevalence in wastewater |
| 218 | Vivek, 2011 | Hepatitis e virus detection and characterization in sewage from Vellore, South India. | No data on HEV prevalence in wastewater |
| 219 | Vonstille, 1993 | Hepatitis A epidemics from utility sewage in Ocoee, Florida. | No data on HEV prevalence in wastewater |
| 220 | Wang, 2020 | Hepatitis E virus genotype 3 strains and a plethora of other viruses detected in raw and still in tap water. | No data on HEV prevalence in wastewater |
| 221 | Wang, 2018 | Differential removal of human pathogenic viruses from sewage by conventional and ozone treatments. | No data on HEV prevalence in wastewater |
| 222 | Winn, 1999 | Enterically transmitted hepatitis - Hepatitis A and E viruses. | Review |
| 223 | Wurtzer, 2021 | Viral infectious diseases seen through wastewater. | Full text or abstract not found |
| 224 | Yoder, 2008 | Surveillance for waterborne disease and outbreaks associated with drinking water and water not intended for drinking--United States, 2005-2006. | No data on HEV prevalence in wastewater |
| 225 | Yugo, 2013 | Hepatitis E virus: Foodborne, waterborne and zoonotic transmission. | Review |
| 226 | Zhou, 2015 | Source identification of bacterial and viral pathogens and their survival/fading in the process of wastewater treatment, reclamation, and environmental reuse. | No data on HEV prevalence in wastewater |

**Table S4:** Characteristics of included studies

| **Characteristics** | **Overall**  **n=88** (%) | **Untreated wastewater**  **n=40** (%) | **Treated wastewater**  **n=11** (%) | **Surface water n=21** (%) | **Drinking water n=10** (%) | **Groundwater n=1** (%) | **Others**  **n=5** (%) |
| --- | --- | --- | --- | --- | --- | --- | --- |
| **Year of publication; range** | 1995-2021 | 1998-2020 | 2013-2020 | 2009-2021 | 1995-2020 | 2020 | 2000-2020 |
|  |  |  |  |  |  |  |  |
| **Period of sampling; range** | 1989-2019 | 1989-2019 | 2000-2019 | 2000-2019 | 2000-2019 | 2015-2016 | 2011-2017 |
|  |  |  |  |  |  |  |  |
| **Starting volume for concentration (mL); range** | 10-100000 | 10-100000 | 35-100000 | 180-100000 | 20-100000 | 500-1000 | 25-10000 |
|  |  |  |  |  |  |  |  |
| **Concentration data (genome copies /L); range** | 0-5.8 x 10^8^ | 6.9 x 10^1^-5.8 x 10^8^ | 4 x 10^2^ to 1 x 10^3^ | 0-1.0 x 10^5^ | 0-8.9 x 10^3^ |  |  |
| **Method for concentration** |  |  |  |  |  |  |  |
| Polyethylene glycol precipitation | 15 (17.1) | 8 (20.0) | 3 (27.3) | 3 (14.3) |  |  | 1 (20.0) |
| Ultracentrifugation | 10 (11.4) | 7 (17.5) | 1 (9.1) | 2 (9.5) |  |  |  |
| Tangential flow filtration | 8 (9.1) | 2 (5.0) | 1 (9.1) | 3 (14.3) | 2 (20.0) |  |  |
| Filtration method | 7 (8.0) | 4 (10.0) |  | 1 (4.8) | 2 (20.0) |  |  |
| Filtration method, Polyethylene glycol precipitation | 6 (6.8) | 3 (7.5) | 1 (9.1) | 2 (9.5) |  |  |  |
| Adsorption-elution method | 4 (4.6) | 2 (5.0) | 1 (9.1) |  |  |  | 1 (20.0) |
| Filtration method, Organic flocculation | 4 (4.6) |  |  | 2 (9.5) | 1 (10.0) |  | 1 (20.0) |
| Polyethylene glycol precipitation, Tangential flow filtration | 4 (4.6) | 2 (5.0) | 1 (9.1) | 1 (4.8) |  |  |  |
| Skimmed Milk Flocculation | 4 (4.6) |  |  | 1 (4.8) | 1 (10.0) | 1 (100) | 1 (20.0) |
| Polyethylene glycol precipitation, UltraAluminum hydroxide precipitation | 3 (3.4) | 1 (2.5) | 1 (9.1) |  | 1 (10.0) |  |  |
| Polyethylene glycol precipitation, Ultrafiltration method | 3 (3.4) | 1 (2.5) | 1 (9.1) | 1 (4.8) |  |  |  |
| Adsorption-elution method, Ultrafiltration method | 2 (2.3) |  |  | 2 (9.5) |  |  |  |
| Filtration method, Skimmed-milk flocculation method | 2 (2.3) | 1 (2.5) | 1 (9.1) |  |  |  |  |
| Flocculation with skimmed milk, Polyethylene glycol precipitation, Tangential flow filtration | 2 (2.3) | 1 (2.5) |  |  | 1 (10.0) |  |  |
| Adsorption-elution method, Filtration method | 1 (1.1) |  |  |  | 1 (10.0) |  |  |
| Ultrafiltration method | 1 (1.1) |  |  | 1 (4.8) |  |  |  |
| Unclear/ Not reported | 12 (13.6) | 8 (20.0) |  | 2 (9.5) | 1 (10.0) |  | 1 (20.0) |
|  |  |  |  |  |  |  |  |
| **Method for RNA extraction** |  |  |  |  |  |  |  |
| QIAamp Viral RNA mini kit | 27 (30.7) | 11 (27.5) | 2 (18.2) | 7 (33.3) | 5 (50.0) | 1 (100) | 1 (20.0) |
| NucliSENS easyMAG/miniMAG | 23 (26.1) | 11 (27.5) | 5 (45.5) | 6 (28.6) |  |  | 1 (20.0) |
| Guanidinium thiocyanate | 6 (6.8) | 3 (7.5) |  | 1 (4.8) | 1 (10.0) |  | 1 (20.0) |
| MagNA Pure Compact Nucleic Acid Isolation Kit | 3 (3.4) |  | 1 (9.1) | 2 (9.5) |  |  |  |
| NucleoSpin RNA virus kit, NucliSENS easyMAG/miniMAG | 3 (3.4) | 1 (2.5) | 1 (9.1) |  | 1 (10.0) |  |  |
| DNeasy Blood and Tissue kit | 2 (2.3) | 1 (2.5) | 1 (9.1) |  |  |  |  |
| InnuPREP Viral RNA Kit | 2 (2.3) | 2 (5.0) |  |  |  |  |  |
| NucleoSpin RNA virus kit | 2 (2.3) | 1 (2.5) |  |  |  |  | 1 (20.0) |
| NucliSENS easyMAG/miniMAG, QIAamp Viral RNA mini kit | 2 (2.3) |  |  | 1 (4.8) | 1 (10.0) |  |  |
| Phenol-chloroform | 2 (2.3) | 1 (2.5) |  |  | 1 (10.0) |  |  |
| TRIzOL reagent | 2 (2.3) | 2 (5.0) |  |  |  |  |  |
| BIOZOL Total RNA Extraction reagent | 1 (1.1) | 1 (2.5) |  |  |  |  |  |
| High Pure Viral Nucleic Acid Kit | 1 (1.1) |  |  | 1 (4.8) |  |  |  |
| Pure Link Viral DNA/RNA mini kit | 1 (1.1) |  |  |  |  |  | 1 (20.0) |
| Pure Link Viral DNA/RNA mini kit, TRIzOL reagent | 1 (1.1) |  |  | 1 (4.8) |  |  |  |
| RNeasy mini kit | 1 (1.1) |  |  | 1 (4.8) |  |  |  |
| RTP DNA/RNA Virus Mini Kit | 1 (1.1) |  |  | 1 (4.8) |  |  |  |
| Tri-reagent | 1 (1.1) | 1 (2.5) |  |  |  |  |  |
| UltraPureTM RNA Kit | 1 (1.1) | 1 (2.5) |  |  |  |  |  |
| Unclear/ Not reported1 | 6 (6.8) | 4 (10.0) | 1 (9.1) |  | 1 (10.0) |  |  |
|  |  |  |  |  |  |  |  |
| **Process control?** |  |  |  |  |  |  |  |
| Yes | 42 (47.7) | 16 (40.0) | 8 (72.7) | 12 (57.1) | 4 (40.0) |  | 2 (40.0) |
| Unclear/ Not reported | 46 (52.3) | 24 (60.0) | 3 (27.3) | 9 (42.9) | 6 (60.0) | 1 (100) | 3 (60.0) |
|  |  |  |  |  |  |  |  |
| **Detection/typing region** |  |  |  |  |  |  |  |
| ORF1 | 13 (14.8) | 7 (17.5) | 1 (9.1) | 4 (19.1) | 1 (10.0) |  |  |
| ORF1, ORF2 | 12 (13.6) | 6 (15.0) | 1 (9.1) | 5 (23.8) |  |  |  |
| ORF1, ORF2, ORF3 | 4 (4.6) | 3 (7.5) |  | 1 (4.8) |  |  |  |
| ORF2 | 11 (12.5) | 6 (15.0) | 1 (9.1) | 3 (14.3) | 1 (10.0) |  |  |
| ORF2, ORF3 | 5 (5.7) | 2 (5.0) |  |  | 1 (10.0) |  | 2 (40.0) |
| ORF3 | 1 (1.1) |  |  | 1 (4.8) |  |  |  |
| Unclear/ Not reported | 42 (47.7) | 16 (40.0) | 8 (72.7) | 7 (33.3) | 7 (70.0) | 1 (100) | 3 (60.0) |
|  |  |  |  |  |  |  |  |
| **Genotype detected (number)** |  |  |  |  |  |  |  |
| HEV-1 | 1 (1.1) |  |  | 1 (4.8) |  |  |  |
| HEV-1 (1) | 1 (1.1) | 1 (2.5) |  |  |  |  |  |
| HEV-1 (10) | 1 (1.1) | 1 (2.5) |  |  |  |  |  |
| HEV-1 (18); HEV-3 (1) | 1 (1.1) | 1 (2.5) |  |  |  |  |  |
| HEV-1 (6) | 1 (1.1) | 1 (2.5) |  |  |  |  |  |
| HEV-1 (8) | 1 (1.1) | 1 (2.5) |  |  |  |  |  |
| HEV-3 (1) | 5 (5.7) | 3 (7.5) |  | 2 (9.5) |  |  |  |
| HEV-3 (14) | 1 (1.1) | 1 (2.5) |  |  |  |  |  |
| HEV-3 (15) | 1 (1.1) | 1 (2.5) |  |  |  |  |  |
| HEV-3 (2) | 5 (5.7) | 2 (5.0) |  | 3 (14.3) |  |  |  |
| HEV-3 (2) and HEV-1 (1) | 1 (1.1) | 1 (2.5) |  |  |  |  |  |
| HEV-3 (25) and HEV-1 (13) | 1 (1.1) | 1 (2.5) |  |  |  |  |  |
| HEV-3 (3) | 5 (5.7) | 3 (7.5) |  | 1 (4.8) |  |  | 1 (20.0) |
| HEV-3 (4) | 1 (1.1) | 1 (2.5) |  |  |  |  |  |
| HEV-3 (5) | 3 (3.4) | 2 (5.0) |  | 1 (4.8) |  |  |  |
| HEV-3 (56); HEV-1 (18) | 1 (1.1) | 1 (2.5) |  |  |  |  |  |
| HEV-3 (6) | 1 (1.1) | 1 (2.5) |  |  |  |  |  |
| HEV-4 (2) | 1 (1.1) | 1 (2.5) |  |  |  |  |  |
| Unclear/ Not reported | 56 (63.6) | 17 (42.5) | 11 (100) | 13 (61.9) | 10 (100) | 1 (100) | 4 (80.0) |
|  |  |  |  |  |  |  |  |
| **Study Design** |  |  |  |  |  |  |  |
| Cross-sectional | 88 (100) | 40 (100) | 11 (100) | 21 (100) | 10 (100) | 1 (100) | 5 (100) |
|  |  |  |  |  |  |  |  |
| **Sampling** |  |  |  |  |  |  |  |
| Non probabilistic | 64 (72.7) | 28 (70.0) | 8 (72.7) | 15 (71.4) | 8 (80.0) | 1 (100) | 4 (80.0) |
| Probabilistic | 24 (27.3) | 12 (30.0) | 3 (27.3) | 6 (28.6) | 2 (20.0) |  | 1 (20.0) |
| **Number of sites** |  |  |  |  |  |  |  |
| Monocenter | 46 (52.3) | 21 (52.5) | 7 (63.6) | 10 (47.6) | 5 (50.0) |  | 3 (60.0) |
| Multicenter | 41 (46.6) | 18 (45.0) | 4 (36.4) | 11 (52.4) | 5 (50.0) | 1 (100) | 2 (40.0) |
| Nationally representative | 1 (1.1) | 1 (2.5) |  |  |  |  |  |
|  |  |  |  |  |  |  |  |
| **Rural/Urban** |  |  |  |  |  |  |  |
| Urban | 34 (38.6) | 14 (35.0) | 3 (27.3) | 8 (38.1) | 6 (60.0) | 1 (100) | 2 (40.0) |
| Rural | 5 (5.7) | 2 (5.0) |  | 1 (4.8) |  |  | 2 (40.0) |
| Urban/Rural | 7 (8.0) | 5 (12.5) |  | 1 (4.8) | 1 (10.0) |  |  |
| Unclear/Not reported | 42 (47.7) | 19 (47.5) | 8 (72.7) | 11 (52.4) | 3 (30.0) |  | 1 (20.0) |
|  |  |  |  |  |  |  |  |
| **Timing of samples collection** |  |  |  |  |  |  |  |
| Prospectively | 87 (98.9) | 39 (97.5) | 11 (100) | 21 (95.2) | 10 (100) | 1 (100) | 5 (100) |
| Retroprospectively | 1 (1.1) | 1 (2.5) |  |  |  |  |  |
|  |  |  |  |  |  |  |  |
| **Countries** |  |  |  |  |  |  |  |
| Italy | 18 (20.5) | 8 (20.0) | 3 (27.3) | 6 (28.6) | 1 (10.0) |  |  |
| Spain | 10 (11.4) | 4 (10.0) | 1 (9.1) | 1 (4.8) | 2 (20.0) | 1 (100) | 1 (20.0) |
| India | 9 (10.2) | 4 (10.0) |  | 1 (4.8) | 4 (40.0) |  |  |
| France | 7 (8.0) | 3 (7.5) | 2 (18.2) | 1 (4.8) |  |  | 1 (20.0) |
| Germany | 5 (5.7) | 2 (5.0) | 1 (9.1) | 1 (4.8) |  |  | 1 (20.0) |
| Egypt | 4 (4.6) | 3 (7.5) | 1 (9.1) |  |  |  |  |
| United Kingdom | 4 (4.6) | 2 (5.0) | 1 (9.1) | 1 (4.8) |  |  |  |
| Argentina | 3 (3.4) | 1 (2.5) |  | 2 (9.5) |  |  |  |
| Portugal | 3 (3.4) | 1 (2.5) |  | 1 (4.8) | 1 (10.0) |  |  |
| Uganda | 3 (3.4) |  |  | 1 (4.8) | 1 (10.0) |  | 1 (20.0) |
| Colombia | 2 (2.3) | 1 (2.5) |  |  | 1 (10.0) |  |  |
| Slovenia | 2 (2.3) |  | 1 (9.1) | 1 (4.8) |  |  |  |
| Sweden | 2 (2.3) | 1 (2.5) | 1 (9.1) |  |  |  |  |
| Tunisia | 2 (2.3) | 2 (5.0) |  |  |  |  |  |
| Brazil | 1 (1.1) |  |  | 1 (4.8) |  |  |  |
| China | 1 (1.1) | 1 (2.5) |  |  |  |  |  |
| Greece | 1 (1.1) | 1 (2.5) |  |  |  |  |  |
| Israel | 1 (1.1) | 1 (2.5) |  |  |  |  |  |
| Japan | 1 (1.1) | 1 (2.5) |  |  |  |  |  |
| Kenya | 1 (1.1) |  |  | 1 (4.8) |  |  |  |
| Netherlands | 1 (1.1) |  |  | 1 (4.8) |  |  |  |
| New Zealand | 1 (1.1) |  |  | 1 (4.8) |  |  |  |
| Pakistan | 1 (1.1) | 1 (2.5) |  |  |  |  |  |
| Philippines | 1 (1.1) |  |  | 1 (4.8) |  |  |  |
| Romania | 1 (1.1) | 1 (2.5) |  |  |  |  |  |
| Switzerland | 1 (1.1) | 1 (2.5) |  |  |  |  |  |
| Thailand | 1 (1.1) |  |  |  |  |  | 1 (20.0) |
| United States of America | 1 (1.1) | 1 (2.5) |  |  |  |  |  |
|  |  |  |  |  |  |  |  |
| **WHO Region** |  |  |  |  |  |  |  |
| Europe | 56 (63.6) | 25 (62.5) | 10 (90.9) | 13 (61.9) | 4 (40.0) | 1 (100) | 3 (60.0) |
| South-East Asia | 10 (11.4) | 4 (10.0) |  | 1 (4.8) | 4 (40.0) |  | 1 (20.0) |
| America | 7 (8.0) | 3 (7.5) |  | 3 (14.3) | 1 (10.0) |  |  |
| Eastern Mediterranean | 7 (8.0) | 6 (15.0) | 1 (9.1) |  |  |  |  |
| Africa | 4 (4.6) |  |  | 2 (9.5) | 1 (10.0) |  | 1 (20.0) |
| Western Pacific | 4 (4.6) | 2 (5.0) |  | 2 (9.5) |  |  |  |
|  |  |  |  |  |  |  |  |
| **UNSD Region** |  |  |  |  |  |  |  |
| Southern Europe | 34 (38.6) | 14 (35.0) | 5 (45.5) | 9 (42.9) | 4 (40.0) | 1 (100) | 1 (20.0) |
| Western Europe | 14 (15.9) | 6 (15.0) | 3 (27.3) | 3 (14.3) |  |  | 2 (40.0) |
| Southern Asia | 10 (11.4) | 5 (12.5) |  | 1 (4.8) | 4 (40.0) |  |  |
| Northern Africa | 6 (6.8) | 5 (12.5) | 1 (9.1) |  |  |  |  |
| Northern Europe | 6 (6.8) | 3 (7.5) | 2 (18.2) | 1 (4.8) |  |  |  |
| South America | 6 (6.8) | 2 (5.0) |  | 3 (14.3) | 1 (10.0) |  |  |
| Eastern Africa | 4 (4.6) |  |  | 2 (9.5) | 1 (10.0) |  | 1 (20.0) |
| Eastern Asia | 2 (2.3) | 2 (5.0) |  |  |  |  |  |
| Southeastern Asia | 2 (2.3) |  |  | 1 (4.8) |  |  | 1 (20.0) |
| Eastern Europe | 1 (1.1) | 1 (2.5) |  |  |  |  |  |
| Northern America | 1 (1.1) | 1 (2.5) |  |  |  |  |  |
| Oceania | 1 (1.1) |  |  | 1 (4.8) |  |  |  |
| Western Asia | 1 (1.1) | 1 (2.5) |  |  |  |  |  |
|  |  |  |  |  |  |  |  |
| **Country income level** |  |  |  |  |  |  |  |
| High-income economies | 59 (67.1) | 27 (67.5) | 10 (90.9) | 14 (66.7) | 4 (40.0) | 1 (100) | 3 (60.0) |
| Lower-middle income economies | 18 (20.5) | 10 (25.0) | 1 (9.1) | 3 (14.3) | 4 (40.0) |  |  |
| Upper-middle-income economies | 8 (9.1) | 3 (7.5) |  | 3 (14.3) | 1 (10.0) |  | 1 (20.0) |
| Low-income economies | 3 (3.4) |  |  | 1 (4.8) | 1 (10.0) |  | 1 (20.0) |
|  |  |  |  |  |  |  |  |
| **Environmental matrices** |  |  |  |  |  |  |  |
| Sewage sample | 21 (23.9) | 21 (52.5) |  |  |  |  |  |
| Surface water | 8 (9.1) |  |  | 21 (100) |  |  |  |
| River water | 7 (8.0) |  |  | 7 (33.3) |  |  |  |
| Sewage effluent | 7 (8.0) |  | 7 (63.6) |  |  |  |  |
| Drinking water | 6 (6.8) |  |  |  | 10 (100) |  |  |
| Sewage influent | 5 (5.7) | 5 (12.5) |  |  |  |  |  |
| Wastewater sample | 4 (4.6) | 4 (10.0) |  |  |  |  |  |
| Raw sewage | 3 (3.4) | 3 (7.5) |  |  |  |  |  |
| Treated sewage | 2 (2.3) |  | 2 (18.2) |  |  |  |  |
| Bore well water | 1 (1.1) |  |  |  | 1 (10.0) |  |  |
| Drink water | 1 (1.1) |  |  |  | 1 (10.0) |  |  |
| Drinking water treatment plants, Influent, Effluent | 1 (1.1) |  |  |  | 1 (10.0) |  |  |
| Effluents of a pig slaughterhouse | 1 (1.1) |  |  |  |  |  | 1 (20.0) |
| Flood water | 1 (1.1) |  |  |  |  |  | 1 (20.0) |
| Freshwater lake with variations in chemical contaminants | 1 (1.1) |  |  | 1 (4.8) |  |  |  |
| Grey water | 1 (1.1) |  |  |  |  |  | 1 (20.0) |
| Groundwater | 1 (1.1) |  |  |  |  | 1 (100) |  |
| Inlet from sewage treatment plants | 1 (1.1) | 1 (2.5) |  |  |  |  |  |
| Irrigation water | 1 (1.1) |  |  |  |  |  | 1 (20.0) |
| Outlet from sewage treatment plants | 1 (1.1) |  | 1 (9.1) |  |  |  |  |
| Pipe water samples | 1 (1.1) |  |  |  | 1 (10.0) |  |  |
| Raw wastewater | 1 (1.1) | 1 (2.5) |  |  |  |  |  |
| Reservoir water, Reclaimed water | 1 (1.1) |  |  |  |  |  | 1 (20.0) |
| River samples | 1 (1.1) |  |  | 1 (4.8) |  |  |  |
| Seawater | 1 (1.1) |  |  | 1 (4.8) |  |  |  |
| Seawater samples | 1 (1.1) |  |  | 1 (4.8) |  |  |  |
| Sewage samples from the inlets | 1 (1.1) | 1 (2.5) |  |  |  |  |  |
| Sewage samples, Sewerage water | 1 (1.1) | 1 (2.5) |  |  |  |  |  |
| Surface water and from its tributaries | 1 (1.1) |  |  | 1 (4.8) |  |  |  |
| Untreated sewage | 1 (1.1) | 1 (2.5) |  |  |  |  |  |
| Wastewater | 1 (1.1) | 1 (2.5) |  |  |  |  |  |
| Wastewater treatment plants, Effluent | 1 (1.1) |  | 1 (9.1) |  |  |  |  |
| Wastewater treatment plants, Influent | 1 (1.1) | 1 (2.5) |  |  |  |  |  |
| Water samples | 1 (1.1) |  |  | 1 (4.8) |  |  |  |
|  |  |  |  |  |  |  |  |
| **HEV detection method** |  |  |  |  |  |  |  |
| Real-time RT-PCR | 60 (68.2) | 24 (60.0) | 11 (100) | 14 (66.7) | 7 (70.0) | 1 (100) | 3 (60.0) |
| Conventional RT-PCR | 28 (31.8) | 16 (40.0) |  | 7 (28.6) | 3 (30.0) |  | 2 (40.0) |
|  |  |  |  |  |  |  |  |
| **Risk of bias** |  |  |  |  |  |  |  |
| Moderate risk of bias | 74 (84.1) | 32 (80.0) | 9 (81.8) | 17 (81.0) | 10 (100) | 1 (100) | 5 (100) |
| Low risk of bias | 14 (15.9) | 8 (20.0) | 2 (18.2) | 4 (19.1) |  |  |  |

**Table S5:** Detailed results of meta-analysis results for prevalence of hepatitis E virus in various water matrices.

|  | **Prevalence (%) [95%CI]** | **95% Prediction interval** | **Number of studies** | **Number of samples** | **H**  **[95%CI]** | **I²**  **[95%CI]** | **P hetero-geneity** | **P difference subtypes** |
| --- | --- | --- | --- | --- | --- | --- | --- | --- |
| **HEV prevalence in water matrices** |  |  |  |  |  |  |  |  |
| **Sampling** |  |  |  |  |  |  |  | 0.716 |
| Non probabilistic | 10.2 [6.2-14.8] | 0-56.2 | 64 | 5298 | 4.4 [4.1-4.8] | 94.9 [94-95.6] | <0.001 |  |
| Probabilistic | 9.1 [3.2-17.2] | 0-61.6 | 23 | 2178 | 5.1 [4.6-5.8] | 96.2 [95.2-97] | <0.001 |  |
|  |  |  |  |  |  |  |  |  |
| **Rural/Urban** |  |  |  |  |  |  |  | 0.064 |
| Rural | 2.2 [0-11] | 0-52.4 | 5 | 345 | 2.9 [2-4.2] | 88 [74.5-94.3] | <0.001 |  |
| Urban | 12 [5.9-19.7] | 0-67.1 | 34 | 3139 | 5 [4.5-5.5] | 96 [95.1-96.7] | <0.001 |  |
|  |  |  |  |  |  |  |  |  |
| **Countries** |  |  |  |  |  |  |  | <0.001 |
| Argentina | 2.6 [0.3-6.4] | 0-71.1 | 3 | 268 | 1.2 [1-2.2] | 35 [0-79] | 0.214 |  |
| Brazil | 0 [0-0.7] | NA | 1 | 250 | NA | NA | 1 |  |
| China | 1.3 [0-3.9] | NA | 1 | 152 | NA | NA | 1 |  |
| Colombia | 19.9 [10.4-31.3] | NA | 2 | 60 | 1 NA | 0 NA | 0.534 |  |
| Egypt | 4.5 [0-27.9] | 0-100 | 3 | 136 | 3.6 [2.3-5.7] | 92.4 [81-97] | <0.001 |  |
| France | 15 [0.9-38.5] | 0-96.4 | 7 | 374 | 4.6 [3.6-5.8] | 95.2 [92.2-97] | <0.001 |  |
| Germany | 39.8 [10.8-73.1] | 0-100 | 5 | 322 | 5.8 [4.5-7.5] | 97 [95.1-98.2] | <0.001 |  |
| Greece | 0 [0-3.6] | NA | 1 | 48 | NA | NA | 1 |  |
| India | 11.9 [0.9-30.9] | 0-89.3 | 9 | 1461 | 8.1 [7-9.4] | 98.5 [98-98.9] | <0.001 |  |
| Israel | 8.3 [4.5-13] | NA | 1 | 169 | NA | NA | 1 |  |
| Italy | 6.1 [3.6-9] | 0.3-16.4 | 18 | 2565 | 1.7 [1.3-2.1] | 63.3 [39.1-77.9] | <0.001 |  |
| Japan | 0 [0-13.9] | NA | 1 | 12 | NA | NA | 1 |  |
| Kenya | 3.2 [1.2-6.1] | NA | 1 | 216 | NA | NA | 1 |  |
| Netherlands | 16.7 [0.4-43.9] | NA | 1 | 12 | NA | NA | 1 |  |
| New Zealand | 3.7 [0.8-8.2] | NA | 1 | 109 | NA | NA | 1 |  |
| Pakistan | 40.7 [30.5-51.3] | NA | 1 | 86 | NA | NA | 1 |  |
| Philippines | 25 [3.9-53.9] | NA | 1 | 12 | NA | NA | 1 |  |
| Portugal | 45.1 [1.6-94.5] | 0-100 | 3 | 123 | 6.3 [4.5-8.8] | 97.5 [95.2-98.7] | <0.001 |  |
| Romania | 10 [0.2-27.8] | NA | 1 | 20 | NA | NA | 1 |  |
| Slovenia | 1.7 [0-7.2] | NA | 2 | 72 | 1 NA | 0 NA | 0.688 |  |
| Spain | 5.5 [0-17.3] | 0-62.5 | 10 | 325 | 3.1 [2.4-3.9] | 89.5 [82.8-93.6] | <0.001 |  |
| Sweden | 35.8 [0-100] | NA | 2 | 37 | 6.3 [4-10] | 97.5 [93.8-99] | <0.001 |  |
| Switzerland | 32.3 [24.3-40.8] | NA | 1 | 124 | NA | NA | 1 |  |
| Thailand | 0 [0-1.7] | NA | 1 | 100 | NA | NA | 1 |  |
| Tunisia | 1.1 [0-3.4] | NA | 2 | 200 | 1 NA | 2.2 NA | 0.312 |  |
| Uganda | 0 [0-3.6] | 0-70.6 | 3 | 52 | 1 [1-3.1] | 0 [0-89.6] | 0.957 |  |
| United Kingdom | 11 [0-48.1] | 0-100 | 4 | 158 | 5.2 [3.8-7] | 96.2 [93-98] | <0.001 |  |
| United States of America | 23.1 [3.6-50.4] | NA | 1 | 13 | NA | NA | 1 |  |
|  |  |  |  |  |  |  |  |  |
| **WHO Region** |  |  |  |  |  |  |  | 0.001 |
| Africa | 1.1 [0-3.3] | 0-7 | 4 | 268 | 1 [1-2.6] | 0 [0-84.7] | 0.813 |  |
| America | 6.5 [0.9-15.4] | 0-45.1 | 7 | 591 | 2.9 [2.1-4] | 88.2 [78.2-93.7] | <0.001 |  |
| Eastern Mediterranean | 6.5 [0-22.7] | 0-81 | 6 | 422 | 4.6 [3.6-6] | 95.3 [92.2-97.2] | <0.001 |  |
| Europe | 12.2 [7.6-17.7] | 0-61 | 56 | 4349 | 4.3 [4-4.7] | 94.7 [93.7-95.5] | <0.001 |  |
| South-East Asia | 9.9 [0.7-26.2] | 0-83.1 | 10 | 1561 | 7.8 [6.8-8.9] | 98.3 [97.8-98.7] | <0.001 |  |
| Western Pacific | 2.6 [0-9.1] | 0-40.4 | 4 | 285 | 1.7 [1-2.9] | 65.2 [0-88.2] | 0.035 |  |
|  |  |  |  |  |  |  |  |  |
| **UNSD Region** |  |  |  |  |  |  |  | <0.001 |
| Eastern Africa | 1.1 [0-3.3] | 0-7 | 4 | 268 | 1 [1-2.6] | 0 [0-84.7] | 0.813 |  |
| Eastern Asia | 0.2 [0-2.4] | NA | 2 | 164 | 1 NA | 0 NA | 0.93 |  |
| Eastern Europe | 10 [0.2-27.8] | NA | 1 | 20 | NA | NA | 1 |  |
| Northern Africa | 2.4 [0-10.2] | 0-46.6 | 5 | 336 | 2.7 [1.8-4] | 86.1 [69.6-93.7] | <0.001 |  |
| Northern America | 23.1 [3.6-50.4] | NA | 1 | 13 | NA | NA | 1 |  |
| Northern Europe | 17.6 [0-52.8] | 0-100 | 6 | 195 | 5 [3.9-6.4] | 96.1 [93.6-97.6] | <0.001 |  |
| Oceania | 3.7 [0.8-8.2] | NA | 1 | 109 | NA | NA | 1 |  |
| South America | 5.3 [0.5-13.5] | 0-43.5 | 6 | 578 | 2.9 [2.1-4.1] | 88.5 [77.5-94.1] | <0.001 |  |
| Southeastern Asia | 5.8 [0-47.7] | NA | 2 | 112 | 3.3 [1.7-6.2] | 90.8 [67.1-97.4] | 0.001 |  |
| Southern Asia | 14.3 [2.3-33.1] | 0-90.2 | 10 | 1547 | 8.1 [7-9.2] | 98.5 [98-98.8] | <0.001 |  |
| Southern Europe | 7.7 [4.3-11.9] | 0-36 | 34 | 3133 | 2.9 [2.6-3.4] | 88.4 [84.9-91.1] | <0.001 |  |
| Western Asia | 8.3 [4.5-13] | NA | 1 | 169 | NA | NA | 1 |  |
| Western Europe | 24.7 [8-46.1] | 0-99.7 | 14 | 832 | 6.2 [5.4-7.1] | 97.4 [96.6-98] | <0.001 |  |
|  |  |  |  |  |  |  |  |  |
| **Country income level** |  |  |  |  |  |  |  | 0.001 |
| High-income economies | 11.9 [7.4-17] | 0-59.6 | 59 | 4483 | 4.2 [3.9-4.6] | 94.4 [93.4-95.2] | <0.001 |  |
| Low-income economies | 0 [0-3.6] | 0-70.6 | 3 | 52 | 1 [1-3.1] | 0 [0-89.6] | 0.957 |  |
| Lower-middle income economies | 9.6 [2.4-20.2] | 0-69 | 17 | 2111 | 6.3 [5.6-7.1] | 97.5 [96.8-98] | <0.001 |  |
| Upper-middle-income economies | 3.2 [0.4-7.7] | 0-24.2 | 8 | 830 | 2.6 [1.9-3.5] | 84.9 [72-91.8] | <0.001 |  |
|  |  |  |  |  |  |  |  |  |
| **Environmental matrice categories** |  |  |  |  |  |  |  | 0.269 |
| Drinking water | 4.7 [0-15.9] | 0-59.9 | 10 | 998 | 4.1 [3.3-5] | 94 [90.9-96] | <0.001 |  |
| Groundwater | 8.3 [0-32.4] | NA | 1 | 12 | NA | NA | 1 |  |
| Others | 4.4 [0-24.4] | 0-91.8 | 6 | 203 | 3.7 [2.8-5] | 92.7 [86.8-95.9] | <0.001 |  |
| Surface water | 7.1 [2.5-13.2] | 0-44.1 | 21 | 1487 | 3.4 [3-4] | 91.6 [88.5-93.8] | <0.001 |  |
| Treated wastewater | 4.4 [0-17] | 0-67.9 | 10 | 376 | 3.6 [2.9-4.5] | 92.3 [87.9-95.1] | <0.001 |  |
| Untreated wastewater | 15.6 [9.8-22.4] | 0-65.3 | 39 | 4400 | 5.1 [4.7-5.6] | 96.2 [95.5-96.8] | <0.001 |  |
|  |  |  |  |  |  |  |  |  |
| **HEV detection method** |  |  |  |  |  |  |  | 0.129 |
| Conventional RT-PCR | 14.2 [8-21.7] | [0-61.2] | 28 | 2404 | 4.3 [3.8-4.8] | 94.6 [93.2-95.7] | <0.001 |  |
| Real-time RT-PCR | 8 [4.2-12.6] | 0-55.6 | 59 | 5072 | 4.7 [4.4-5.1] | 95.6 [94.8-96.2] | <0.001 |  |

**Fig S1:** Funnel chart for publications of the global hepatitis E virus prevalence in water matrices


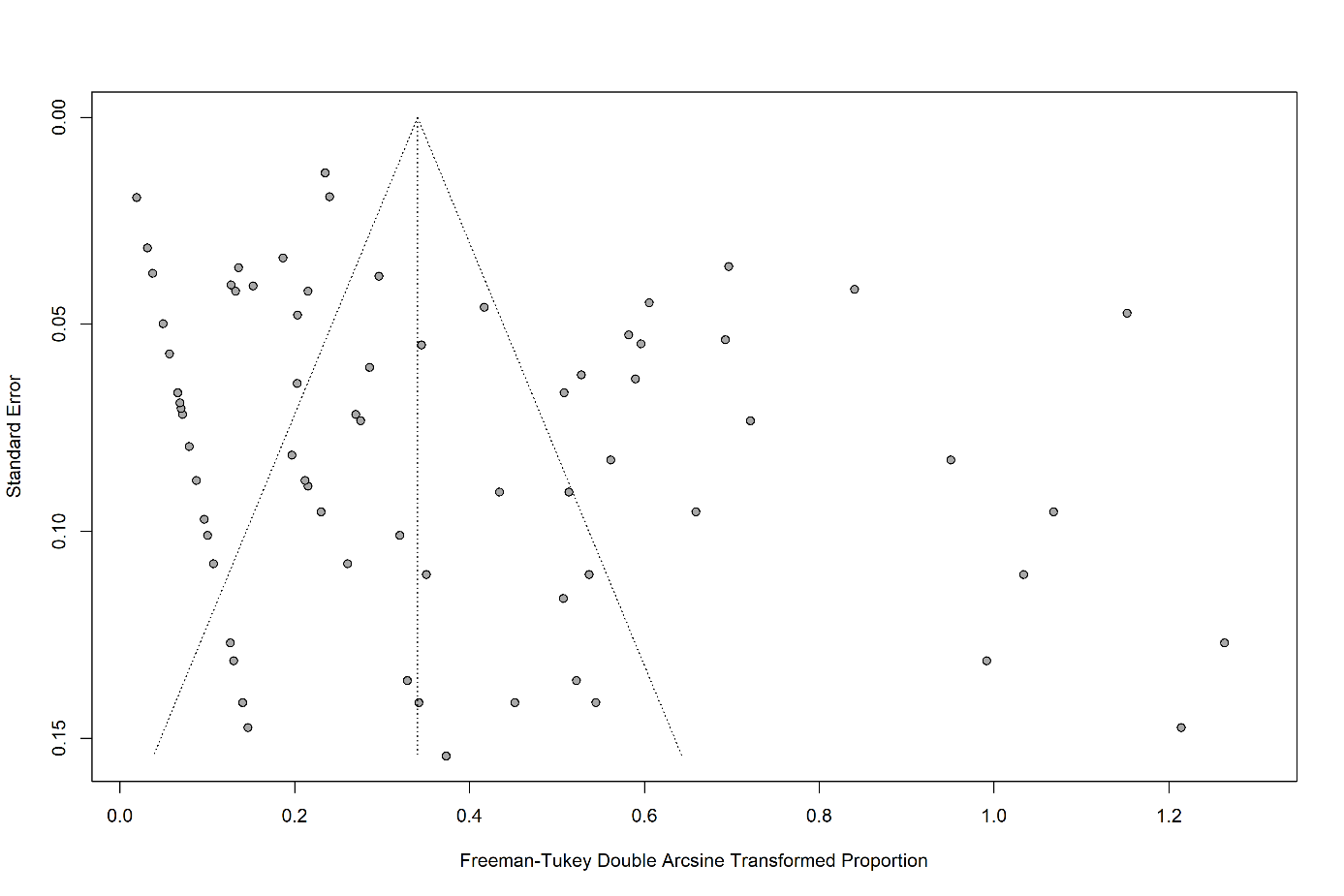


Note: The observation of the asymmetric distribution of the studies on this diagram indicates the existence of publication bias which is confirmed by this P value of the Egger test (P Egger test = 0.020)
